# All identical objects reduce memory load at the late maintenance stage in working memory

**DOI:** 10.1038/s41598-025-00433-4

**Published:** 2025-05-14

**Authors:** Lijing Guo, Hyung-Bum Park, Guofang Ren, Penglan Liu, Ruyi Liu, Dan Nie, Chaoxiong Ye

**Affiliations:** 1 School of Education, Anyang Normal University, Anyang, China; 2 Department of Psychology, University of Jyvaskyla, Jyvaskyla, Finland; 3 Institute for Mind and Biology, University of Chicago, Chicago, USA; 4 Institute of Brain and Psychological Sciences, Sichuan Normal University, Chengdu, China

**Keywords:** Visual short-term memory, Identical objects, Orientation stimulus, Perceptual organization, Contralateral delay activity, Psychology, Human behaviour

## Abstract

Visual working memory (VWM) is critical for temporarily storing and manipulating visual information but is limited in capacity. Previous research suggests that perceptual organization, such as grouping identical objects, may alleviate VWM resource constraints, yet the underlying mechanisms remain unclear. This study investigated whether identical orientation stimuli reduce VWM resource consumption and whether this reduction occurs during encoding or maintenance phase. Using the contralateral delay activity (CDA), an event-related potential (ERP) component that indexes the number of items stored in VWM, we examined three memory conditions: all-same (i.e., four identical orientations), partial-same (i.e., two pairs of identical orientations), and all-different (i.e., four different orientations) conditions. Behavioral results showed improved VWM performance as the number of identical orientations increased, with the highest performance in the all-same condition. ERP results revealed no significant differences in early CDA amplitudes across conditions, suggesting that identical stimuli do not reduce VWM resource consumption during early maintenance phase. However, late CDA amplitudes were significantly reduced in the all-same condition compared to the partial-same and all-different conditions, indicating that the benefits of identical objects emerge during the late maintenance phase. Notably, partially identical stimuli did not reduce VWM load, highlighting the complexity of integrating partially redundant information. These findings highlight the distinct contributions of early consolidation and maintenance phases in optimizing VWM through perceptual grouping and provide evidence that the facilitative effect of identical objects relies on their complete repetition and late-stage cognitive processes.

**Supplementary Information:**

The online version contains supplementary material available at 10.1038/s41598-025-00433-4.

## Introduction

Visual working memory (VWM) is a critical cognitive system responsible for the temporary storage and manipulation of visual information, enabling visual stimuli to remain active even after they are no longer present in the environment^1–3^. As a core component of cognitive functioning, VWM capacity has been shown to predict individual differences in fluid intelligence^4,5^ and performance across a range of cognitive tasks^6^. However, VWM capacity is highly limited, with individuals typically able to actively maintain only about 3–4 simple objects at a time^1,7,8^. These capacity constraints impose significant limitations on broader cognitive abilities. As a result, much research has focused on uncovering the mechanisms underlying these limitations and exploring ways to enhance VWM performance^9–11^. For instance, some studies have explored how reallocating attention during the memory maintenance phase can enhance VWM performance^12–23^. Some other research has examined potential approaches to optimize the encoding process, enabling individuals to store a greater amount of information in VWM^8,24–31^.

In the field of visual perception, researchers have found that, for simple stimuli, bottom-up information about perceptual organization can facilitate perceptual processes and attentional allocation^32–36^. A substantial body of evidence has demonstrated that principles of perceptual organization, particularly those outlined as Gestalt principles, significantly enhance visual perception. Gestalt principles refer to configuration rules that guide the immediate integration of visual stimuli into cohesive percepts before their constituent parts are individually processed^37^. Among these principles, similarity—grouping based on repeated features such as identical colors or orientations—along with proximity and connectedness, has been shown to robustly facilitate visual perception^38^. The mechanisms underlying these facilitative effects of perceptual organization suggest that, during preattentive phases, the visual field is parsed into discrete objects based on principles of perceptual organization^39^. For example, perceptual discrimination tasks remain accurate when stimuli are grouped by Gestalt principles, even under conditions of inattention^40^. Thus, such grouping principles appear to automatically enhance visual perception. However, despite the substantial overlap between perceptual and VWM processes^41^, strong perceptual organization effects observed in visual perception do not necessarily translate into comparable benefits for VWM. As a result, researchers have increasingly sought to determine whether the performance benefits facilitated by perceptual grouping in visual perception extend to VWM^42^.

Previous studies have provided substantial behavioral evidence supporting the role of perceptual organization in improving VWM performance^42–49^. For example, Woodman, et al.^42^ found that in change detection tasks reflecting VWM, participants tended to group items based on Gestalt principles such as proximity and connectedness. When one item in a group was well stored in VWM, the probability of storing other items from the same group also increased. Additionally, VWM performance was better when participants stored multiple identical stimuli compared to multiple distinct ones^45,49,50^.

In real-world environments, individuals frequently encode multiple identical items as a single meaningful unit rather than as separate objects. For example, we may perceive a cluster of flowers of the same color or rows of identical buildings as cohesive groups rather than individual entities. This highlights the practical importance of investigating the mechanisms underlying improved VWM performance when storing identical objects. Previous studies have shown that VWM resource allocation during consolidation involves at least an early phase and a late phase, which operate differently^51–55^. Given the perceptual benefits of identical object organization, one possible explanation for the enhanced performance in VWM observed with identical objects is that their grouping occurs preattentively before the early maintenance phase. When multiple identical items are stored in VWM, they may automatically form a prepackaged “chunk,” creating an early process bias that facilitates the transfer of perceptual representations into VWM and improves performance^45^. This chunking process may also reduce the neural demands associated with VWM. For example, Xu and Chun^56^ used fMRI to show that grouped items were associated with reduced activation in the inferior intraparietal sulcus (IPS) during VWM maintenance compared to ungrouped items. Alternatively, another explanation is that grouping identical objects in VWM may occur during the late maintenance phase instead of during the encoding phase, functioning as a way to compress redundant information and reduce the VWM load. This benefit of grouping in VWM may require time to develop. Xu^49^ provided evidence for this possibility, showing that VWM first selected four identical objects individually. After selection and individuation, object identification processes began, resulting in only one identity representing the four identical items being maintained in the superior IPS. This suggests that the reduced VWM load associated with storing identical objects may emerge as a late-selection process during the maintenance phase. However, it is important to note that behavioral results primarily reflect the final outputs of multiple cognitive processes, including encoding, consolidation, maintenance, retrieval, and comparison. As such, previous behavioral studies are insufficient to differentiate between these two possibilities or to determine the exact cognitive mechanisms responsible for the observed benefits of storing identical objects in VWM.

To address the limitations of behavioral measures, researchers have used high-temporal-resolution techniques, such as event-related potentials (ERPs), to investigate the VWM maintenance process. One key ERP component, the contralateral delay activity (CDA), reflects a sustained negative potential that tracks the number of items actively maintained in VWM. CDA has been widely used to examine VWM processes^57,58^. Typically, as the number of items represented in VWM increases, the amplitude of the CDA also increases; however, once an individual reaches the limit of their VWM capacity, the amplitude of the CDA no longer increases with the number of items to be remembered^6,59^. Compared to traditional behavioral indicator, such as *Cowan’s K*, which estimates the average number of items successfully stored in VWM during a VWM task^60^, CDA provides a real-time tracking of the number of items stored in VWM, occurring before the participant’s response and not influenced by the probe stimuli or the matching decision phase. Moreover, previous studies have shown that CDA primarily tracks the number of VWM representations, rather than being modulated by factors such as the information load^61–63^ or the current focus of spatial attention^64^. As such, CDA amplitude serves as a more direct index of the number of stored VWM items and provides valuable insights into how VWM resources are allocated to storage representations compared to *Cowan’s K*^57^.

Previous research has used the CDA to investigate whether the presence of identical objects enhances VWM performance and to examine the underlying cognitive mechanisms^65–67^. For example, Gao, et al.^65^ asked participants to memorize three memory array conditions: a single color, four identical colors, and four different colors. They found that VWM performance in the four identical colors condition was better than in the four different colors condition. More importantly, the CDA amplitude in the single color and four identical colors conditions was comparable, and both were significantly lower than that in the four different colors condition. These results suggest that when all items within the attentional focus are identical, the number of representations storing in VWM is greatly reduced. Peterson, et al.^66^ further examined whether the benefit of identical objects would persist when only part of the memory array consisted of identical items. In their study, participants were presented with three memory arrays: all-different condition with high load (three color stimuli, each with a different color), all-different condition with low load (two color stimuli, each with a different color), and partial-same condition with high load (three color stimuli, two of which are identical and one different). Their results showed that, compared to the all-different condition with high load, the partial-same condition with high load improved behavioral performance. More importantly, the CDA amplitude in the partial-same condition with high load was comparable to that in the all-different condition with low load, and both were significantly lower than in the all-different condition with high load. These findings suggest that identical colors can alleviate the VWM load, and this benefit is not limited to situations where all items in the visual field are identical. However, a different conclusion was drawn by Shen, et al.^67^, who used a similar paradigm as the study by Peterson, et al.^66^. Participants were presented with three conditions all-different condition with high load (three orientation stimuli, each with a different orientation), all-different condition with low load (two orientation stimuli, each with a different orientation), and partial-same condition with high load (three orientation stimuli, two of which are identical and one different). Although the partial-same condition with high load showed better behavioral performance compared to the all-different condition with high load, CDA results revealed no significant difference in amplitude between the partial-same condition with high load and the all-different condition with high load. Both were significantly higher than the CDA amplitude in the all-different condition with low load. This suggests that, for the orientation stimuli, while identical objects can improve behavioral performance, this benefit may not necessarily reflect reduced VWM load during maintenance. Instead, the behavioral benefit may arise from the influence of identical objects on retrieval or comparison processes in VWM. Taken together, these findings highlight the need for further research to clarify how identical objects are stored in VWM and to resolve the discrepancies observed in previous ERP studies^65–67^.

In the current study, we aimed to provide new evidence regarding the mechanisms underlying the storage of identical objects in VWM. One potential reason for the inconsistencies in previous findings may lie in the type of feature dimension used as stimuli. Certain VWM mechanisms might be specific to a particular feature dimension, meaning findings derived from color stimuli may not generalize to other visual materials. For example, VWM processing for color and orientation stimuli exhibits genuine differences. Alvarez and Cavanagh^68^ proposed that objects defined by boundary features (e.g., shapes, orientations) are processed differently from those defined by surface features (e.g., colors). Stevanovski and Jolicoeur^69^ found that VWM performance for color stimuli was significantly better than for orientation stimuli. Woodman and Vogel^70^ attributed this performance difference to variations in consolidation rates, with color information being consolidated more quickly than orientation information. Additionally, evidence suggests distinct patterns of interaction between VWM number and precision across these feature dimensions. When storing color stimuli, VWM number appears unaffected by changes in precision^62,63^. In contrast, storing orientation stimuli leads to a trade-off, where increased precision results in reduced number^61,71^. A series of studies has further suggested that color stimuli occupy less bandwidth during VWM consolidation, allowing for parallel processing, whereas orientation stimuli and other complex features require larger bandwidth and are often consolidated serially^72–76^. Given the previous evidence that identical colors can reduce VWM load, the current study extends this line of research by further examining whether identical orientation stimuli exhibit similar effects. By focusing on orientation stimuli, this study aimed to shed light on how identical objects are stored in VWM and to address the discrepancies observed in previous studies.

Specifically, this study investigated two key questions regarding how identical objects are stored in VWM. First, we examined whether stimulus uniformity in a memory array of completely identical or partially identical orientation stimuli reduces VWM load. Second, if the storage of identical stimuli indeed reduces VWM load, we aimed to determine whether this reduction occurs during the early maintenance phase—when perceptual representations are transferred to VWM—or whether it arises during the late maintenance phase of VWM representations. To address these questions, we designed an ERP experiment that included three conditions, each requiring participants to remember four items. Importantly, we controlled the content of the items at three different conditions: four different stimuli (i.e., all-different), four identical stimuli (i.e., all-same), or two pairs of stimuli that were same to each other (i.e., partial-same). We measured the CDA as an indicator to track the number of items in VWM. Unlike previous studies^66,67^, which imposed a memory load of three items, we increased the memory load to four stimuli to create a more demanding task. This decision was motivated by earlier findings that the average VWM capacity for undergraduate participants is approximately 2.9 items^77^. By increasing the load, we aimed to encourage participants to adopt strategies for reducing memory difficulty when encountering identical complex stimuli.

Moreover, unlike previous ERP studies^65–67^, all conditions in the current study required participants to store the same number of items (four stimuli). In addition to the all-different condition, we included both all-same and partial-same conditions. Considering the distinct consolidation mechanisms of color and orientation stimuli, as well as inconsistencies in previous ERP findings, it remains unclear whether orientation stimuli reduce VWM resource consumption under all-same or partial-same conditions. The previous ERP study using orientation stimuli did not examine cases where all orientation stimuli were completely identical^67^. Thus, by including both all-same and partial-same conditions, we aimed to determine whether reductions in VWM resource consumption are specific to the unique consolidation patterns of color stimuli or reflect a general phenomenon associated with partial uniformity in stimuli. This allowed us to confirm whether the reduction in VWM resource consumption observed in previous studies^65,66^ was specific to color stimuli or a general phenomenon occurring with partially same stimuli during memory processing.

Here, we propose that our experimental results may align with one of three hypotheses regarding the impact of identical orientation stimuli on the number of items stored in VWM. Firstly, consistent with two previous ERP studies^65,66^, the “Partial Identicality Benefit Effect” hypothesis suggests that the presence of partially same objects within the memory range can reduce VWM resource consumption. According to this, we would expect the CDA amplitude to follow a gradual decrement (i.e., reduced resource consumption) throughout the all-different, partial-same, and all-same conditions in order. Secondly, the “Complete Identicality Benefit Effect” hypothesis posits that, unlike color stimuli, complex stimuli like identical orientations would not always reduce VWM resource consumption unless all items in the visual field are identical. Therefore, the CDA amplitude is expected to be reduced if and only if all four orientation bars are identical in the all-same condition, whereas comparable between all-different and partial-same conditions. Lastly, in line with a previous ERP study^67^, the “No Identicality Benefit” hypothesis states that identical orientations do not lead to a reduction in VWM resource consumption. Consequently, the CDA amplitude would show no significant differences across all memory conditions.

Beyond examining how storing identical objects affects VWM processes, we also aimed to determine whether this effect arises during the early consolidation phase (i.e., before the early maintenance phase) or during the late maintenance phase. By distinguishing between early and late CDA components, we can address this question. Previous studies suggest that adjustments in VWM resource allocation may take time to develop. For instance, by measuring early and late CDA components, Ye, et al.^78^ found that when participants memorized arrays containing distractors, these distractors were initially stored during the early maintenance phase but were excluded from VWM in the late maintenance phase, thereby reducing VWM resource consumption. Similarly, Peterson, et al.^66^ observed that when participants memorized arrays with connected items, a reduction in CDA amplitude—reflecting the connectedness benefit—emerged only during the late maintenance phase. These findings suggest that perceptual organization principles, such as connectedness, influence VWM processes primarily during the late maintenance phase rather than during early consolidation phase. In our study, we hypothesize two possible findings based on early and late CDA result patterns: If the effect of identical objects occurs preattentively before the early maintenance phase, meaning that perceptual representations transferred into VWM already benefit from processing identical objects, the results for early and late CDA components should show similar patterns. Conversely, if the effect arises later during the maintenance phase, such that perceptual representations transferred to VWM during the early consolidation phase do not yet benefit from identical objects, the patterns of early and late CDA components would differ. Specifically, we would expect no effect of identical objects in the early CDA but a significant effect in the late CDA. Therefore, by analyzing CDA components, our study aimed to further clarify the cognitive mechanisms underlying the storage of identical stimuli in VWM.

## Methods

To confirm the effectiveness of the experimental task control, we conducted a behavioral pilot experiment before the formal ERP experiment. Details of this pilot experiment can be found in the Supplementary Materials. In addition to manipulating the three types of memory arrays described earlier, we varied the change angles of the probe to prevent participants from developing fixed expectations about the range of changes. We hypothesized that smaller change angles (e.g., 15°) would make it more challenging for participants to detect changes. The pilot study revealed that when the change angle was very small (15°), there was no significant difference in memory performance between the all-different and partial-same conditions. However, at larger angles (30° and 45°), performance in the all-different condition was worse than in the partial-same and all-same conditions (see Supplementary Materials for details). These findings suggest that the change angle influences the effects of the three memory conditions.

For the formal ERP experiment, we used a similar design to the pilot study to ensure that each memory condition presented a certain level of difficulty, encouraging participants to focus carefully on the stimuli. Specifically, change angles were randomly varied across three levels (15°, 30°, and 60°) whenever a stimulus changed. This setup also helped prevent participants from forming fixed expectations about the change angle.

### Participants

The sample size for the study was determined using a priori power analysis for a 2 (time window: early vs. late) × 3 (memory condition: all-same, partial-same, all-different) within-subjects design, analyzed with repeated-measures analysis of variance (ANOVA) Based on findings from the study by Peterson, et al.^66^, we anticipated a similar effect size of η_p_^2^ = 0.26, aiming for a statistical power of 95% at a significance level of 0.05. The analysis indicated a minimum sample size of 14 participants^79^. Additionally, considering that the study by Peterson, et al.^66^ included 19–22 participants in each experiment, we planned to recruit 20–25 participants for our experiment.

A group of 38 participants from Sichuan Normal University took part in the study and received monetary compensation. The inclusion criteria were 18 years of age or older, self-reported normal color vision, and normal or corrected-to-normal visual acuity. Exclusion criteria included a history of psychiatric disorders, use of nervous system acting drugs, and previous participation in working memory experiments. Thirteen participants were excluded due to excessive electroencephalogram (EEG) artifacts or frequent eye movements. An additional two participants were excluded due to procedural issues: one for inattentiveness resulting in the premature termination of the experiment, and the other due to a program crash during data collection. As a result, 23 participants (18 females, 5 males; average age = 20 years, range = 18–25, SD = ± 0.44) were included in the final analysis. This sample size aligns closely with that of the study by Peterson, et al.^66^. Before the experiment, all participants provided written informed consent. The study was conducted under the Declaration of Helsinki and approved by the Ethics Committee of the Institute of Brain and Psychological Sciences, Sichuan Normal University (Protocol ID: SCNU-221114).

### Stimuli

The procedure of this experiment was programmed using E-Prime. The experimental stimuli were presented on a 23.8-inch LCD display with a resolution of 1280 × 768 and a refresh rate of 60 Hz. The screen background color during the experiment was black (RGB: 0, 0, 0). Each participant was seated approximately 60 centimeters from the screen. Throughout the experiment, a cross-fixation point remained centered on the screen. Memory stimuli and probe stimuli consisted of white (RGB: 225, 225, 225) bars. In the memory array, 8 bars were presented, arranged in a circle around the central cross fixation point with a radius of 5 degree of visual angle (dva). The bars were symmetrically distributed to the left and right of the fixation point. The size of each bar was 1.4 dva × 0.2 dva, with an inter-bar spacing of 2.9 dva and a distance of 3.3 dva from the fixation point. The possible orientation of each bar was randomly selected from 180 possible angles, with any two bars separated by at least 45°. In the test array, one bar appeared at a random position on each side, matching the location of a bar from the memory array. In the probe array, the angles of the bars presented in the memory array were randomly changed by 15°, 30°, or 60° under different conditions.

### Procedure

**Fig. 1:**
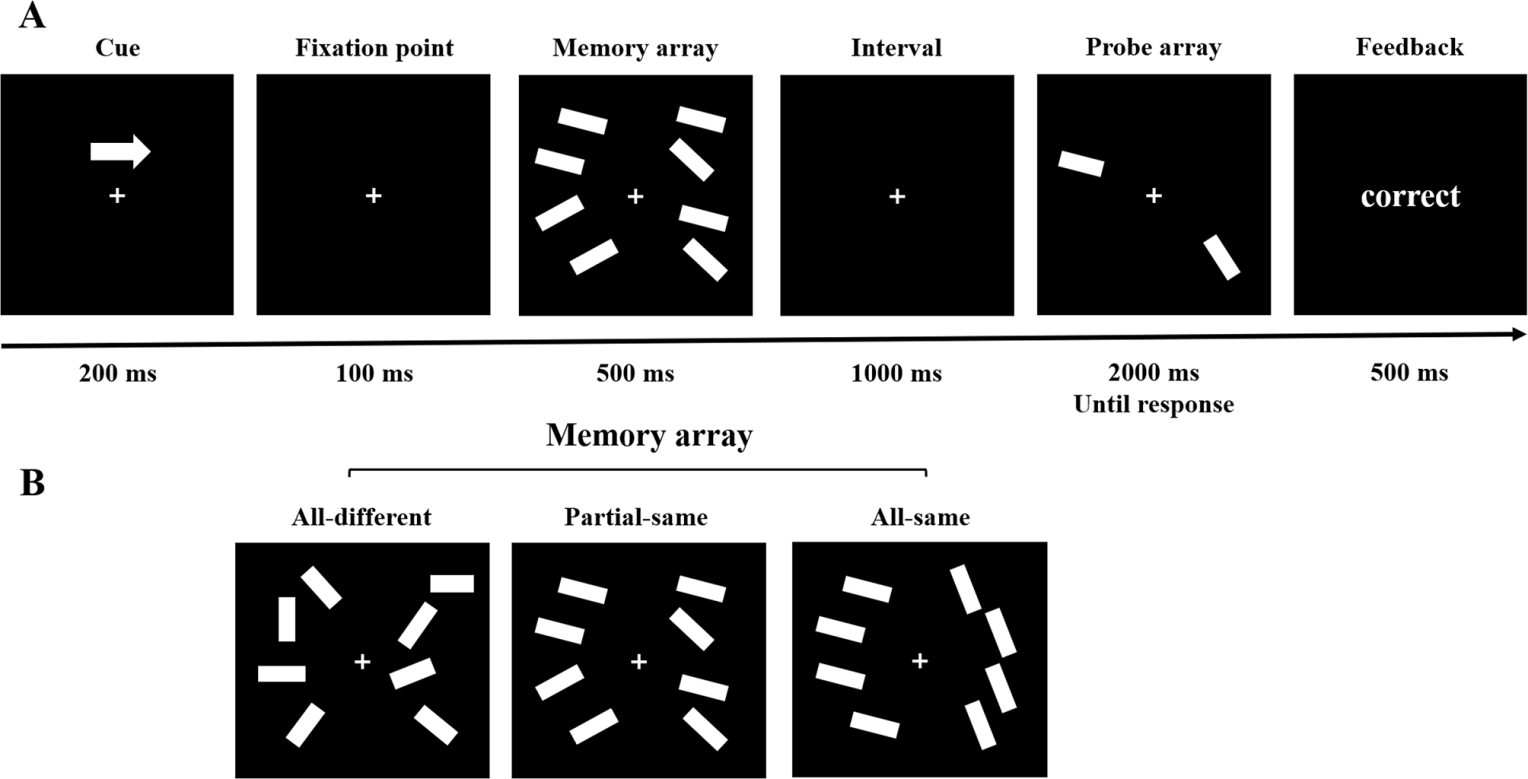
Overview of experimental design. (**A**) Flowchart of the experimental task. (**B**) Three conditions of the memory array: all-same condition; partial-same condition; all-different condition.

The experimental procedure is illustrated in Fig. 1. Throughout the experiment, a fixation cross remained visible at the center of the screen to help participants maintain their focus. Each trial began with an arrow cue phase lasting 200 ms, during which an arrow appeared above the fixation cross, pointing either to the left or right (50% probability for each direction). Participants were instructed to remember the orientations of the bars presented in the cued hemifield while ignoring the bars in the uncued hemifield. Following the offset of the cue, a blank interval of 100 ms was presented, with the fixation cross still displayed in the center of the screen. Next, a memory array containing eight orientation bars was displayed for 500 ms. The memory array was symmetrically divided between the two hemifields, with each hemifield containing four bars. Participants were required to memorize the orientations of the four bars in the cued hemifield. After the memory array disappeared, a blank interval lasting 1000 ms was shown, during which the fixation cross remained visible. Subsequently, a probe array was presented, consisting of one probe stimulus on each side, appearing at a location previously occupied by one of the memory array bars. The probe stimulus had a 50% chance of matching the orientation of the bar at the corresponding location in the memory array and a 50% chance of differing in orientation. If the probe stimulus differed from the corresponding memory bar, its orientation deviated by 15°, 30°, or 60° (each change angle occurred with equal probability, i.e., 1/3). Participants were tasked with judging whether the probe orientation matched the orientation of the corresponding bar in the memory array. They were asked to press the “F” key if the orientations matched and the “J” key if they did not. Each trial ended after the participant’s response or after 2000 ms of screen presentation, whichever came first. Finally, a feedback phase lasting 500 ms was shown, indicating “correct” or “incorrect” based on the participant’s response. The memory array was presented under three conditions: (1) the all-same condition, where all bars within each hemifield had identical orientations; (2) the partial-same condition, where each hemifield contained two pairs of bars with identical orientations; and (3) the all-different condition, where all bars within each hemifield had unique orientations.

Before the formal experiment, participants completed 18 practice trials. The formal experiment consisted of 648 trials in total, with 216 trials randomly assigned to each of the three memory conditions. The experiment lasted approximately one hour, including 17 scheduled breaks to minimize fatigue and ensure data quality. To prevent eye movements from influencing the CDA results, participants were instructed to maintain their gaze on the central fixation cross throughout the experiment, with minimal eye movement allowed.

### Data analysis

#### Electroencephalogram recording and analysis

During the task, we continuously recorded EEG activity using a 62-channel active Ag/AgCl electrode system (Brain Products ACTi Champ) positioned on an elastic cap, according to the International 10–10 system. The ground electrode was placed at FPz. The online reference for the data was set to the vertex (Cz). For the post-recording analyses, the data were re-referenced offline to the average of the bilateral mastoids(TP9/TP10). A horizontal electrooculogram (IO) was recorded by using a referenced electrode pair positioned approximately 1 cm laterally to the outer canthi of right eyes. The impedance at each electrode site was kept below 5 kΩ. The EEG and EOG signals were digitized at a sampling rate of 500 Hz.

The data were processed offline by using MATLAB (2019). The EEG signals were segmented into epochs of 1000 ms duration, starting from 200 ms before the onset of the memory array. A low-pass filter with a cutoff frequency of 30 Hz was applied to the data. Baseline correction was performed by subtracting the average amplitude of the 200-ms peristimulus interval. Trials containing horizontal eye movements, identified by IO amplitudes exceeding ± 60 µV, were excluded from the analysis. Additionally, trials with remaining artifacts exceeding ± 80 µV in amplitude were rejected. Participants with a trial rejection rate higher than 45% were excluded from further analysis. The EEG data from the remaining trials were averaged for each participant and condition, and the averages were time-locked to the onset of the memory array.

Based on our previous CDA studies^80–87^, we selected two pairs of posterior electrode sites (PO7/PO8 and P7/P8) for analysis. For memory condition, contralateral amplitudes were calculated by averaging the activity recorded at the left hemisphere electrode sites when participants were cued to memorize the right side of the memory array. Conversely, activity recorded at the right hemisphere electrode sites was averaged when participants were cued to memorize the left side. Ipsilateral amplitudes were computed by averaging the activity from the left and right hemisphere sites when participants were cued to memorize the left and right sides of the memory array, respectively. The CDA amplitude was determined by subtracting the ipsilateral activity from the contralateral activity within a defined measurement time window following the onset of the memory array.

To facilitate reliable statistical comparisons, we analyzed CDA amplitudes across two equal-length time windows: the early phase (500–650 ms) and the late phase (700–850 ms). These CDA amplitudes (early and late) were analyzed separately for each memory condition and participant. Thus, the primary ERP measures included early CDA amplitude (500–650 ms) and late CDA amplitude (700–850 ms). Additionally, we conducted an analysis of the whole CDA amplitude within the measurement time window of 500–850 ms, detailed in the Supplementary Materials. The results showed that the pattern of the whole CDA amplitude (500–850 ms) closely mirrored that of the late CDA amplitude (700–850 ms). This suggests that the effects observed for the whole time window (500–850 ms) were predominantly driven by contributions from the late maintenance phase. A detailed report of the whole CDA amplitude results (500–850 ms) is available in the Supplementary Materials.

### Statistical analysis

We conducted separate analyses for the behavioral data and the ERP data. For the behavioral results, we used *Cowan’s K* to quantify memory performance^60^. *Cowan’s K* was calculated with the formula: *K* = *N* × (*H* − *F*), where *N* is the set size of memory array (four in our experiment), *H* is the hit rate (the proportion of correct responses when a change was present), and *F* is the false alarm rate (the proportion of incorrect responses when no change was present). Higher *K* values indicate better memory performance. First, we conducted a one-way repeated-measures ANOVA to examine the effect of memory condition (all-same, partial-same, all-different) on *K* values. Planned pairwise comparisons were conducted using two-tailed paired t-tests to compare differences between each pair of conditions (all-same vs. partial-same, all-same vs. all-different, and partial-same vs. all-different). Next, to further investigate the effect of change angle on different memory conditions, we conducted a two-way repeated-measures ANOVA with memory condition (all-same, partial-same, all-different) and change angle (15°, 30°, 60°) as within-subject factors on *K* values. Planned pairwise comparisons for each change angle were conducted with two-tailed paired t-tests to compare differences among the three memory conditions. For the ERP results, we focused on the CDA amplitude. We analyzed early and late CDA amplitudes using a two-way repeated-measures ANOVA with time window (early vs. late) and memory condition (all-same, partial-same, all-different) as within-subject factors. Planned pairwise comparisons within each time window were performed using two-tailed paired t-tests to compare the three memory conditions. The effect size for ANOVA was estimated using the partial eta-squared (η_*p*_^2^ value. JASP (version 0.19) was used to provide *Cohen’ s d*, estimating the effect size for the t-tests, and Bayes factors^88^, showing whether the t-test results supported the alternative hypothesis, thereby providing an odds ratio for the alternative/null hypotheses (values < 0.3 provide evidence for the null hypothesis and values > 3 provide evidence for the alternative hypothesis). All data mentioned in the main text and Supplementary Materials are available through the Open Science Framework at https://osf.io/j6yse/.

## Results

### Behavioral results

#### Effect of memory condition

**Fig. 2:**
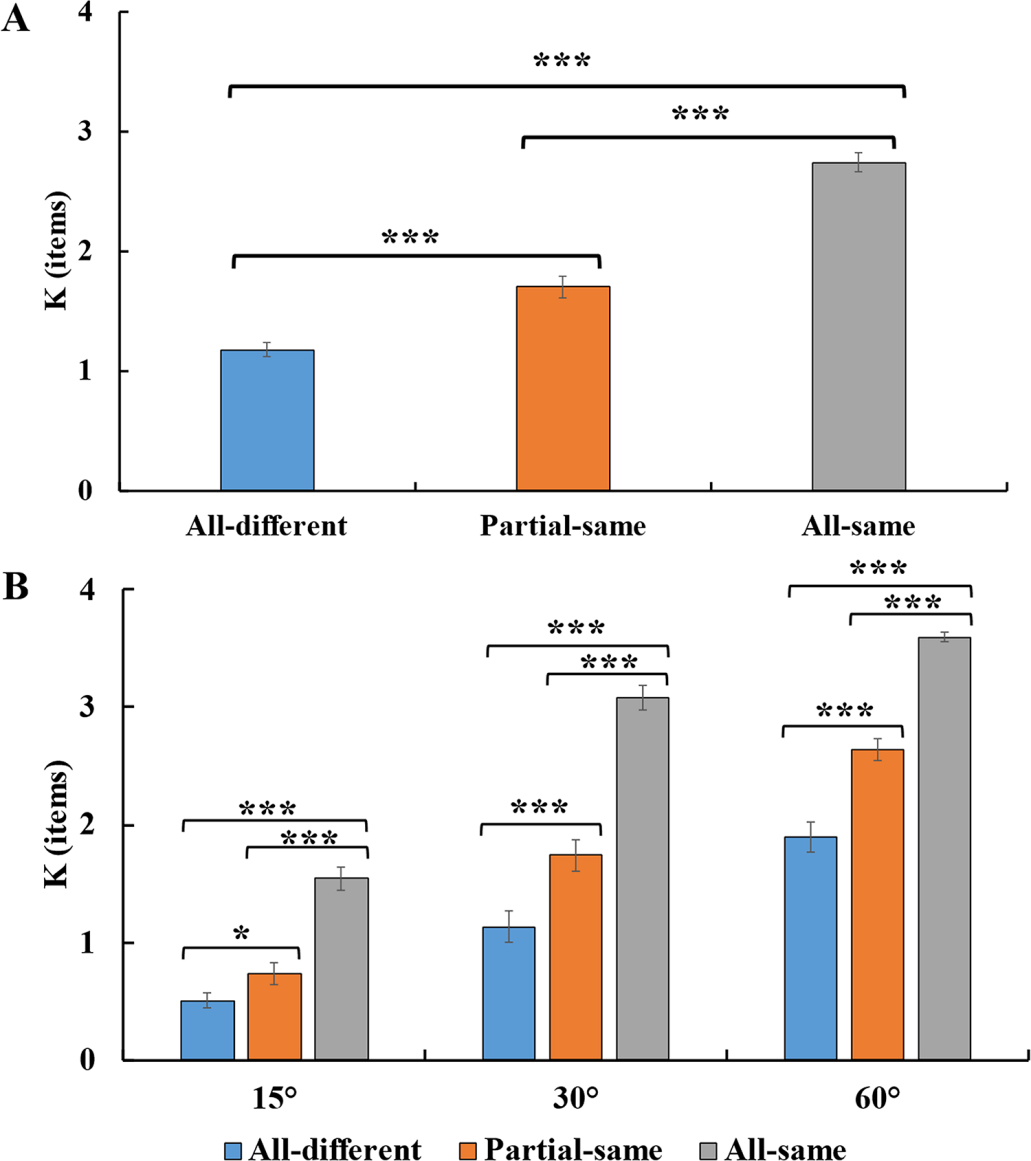
Memory performance across conditions. (**A**): Mean and standard error of the mean for the *K* under different memory array conditions (all-different condition, partial-same condition, and all-same condition). (**B**) *K* results for the three memory arrays under three different conditions of angle change. Error bars indicate SE. * = *p* < .05; *** = *p* < .001.

The mean *K* values for each memory condition (all-same condition vs. partial-same condition vs. all-different condition) is presented in Fig. 2A. The ANOVA revealed a significant main effect of the *K* (mean *K* for the all-same condition, partial-same condition, and all-different condition: 2.74 ± 0.06, 1.70 ± 0.09, 1.18 ± 0.08 items, respectively), *F*(2,44) = 187.06, *p <* .001, η_*p*_^*2*^ = 0.90.

Planned pairwise comparisons revealed that the *K* were significantly lower for the all-different condition than for the all-same condition, *t*(22) = 21.450, *p* < .001, *Cohen’s d =* 4.470, *BF*_*10*_ *>* 1000. *K* was significantly higher in the all-same condition compared to the partial-same condition, *t*(22) = 11.200, *p <* .001, *Cohen’s d =* 2.340, *BF*_*10*_ *>* 1000. The partial-same condition also yielded significantly higher *K* than the all-different condition, *t*(22) = 6.590, *p <* .001, *Cohen’s d =* 1.370, *BF*_*10*_ *>* 1000. These results suggest that the performance of VWM improved with the number of identical orientations increases.

### Effect of change angle across memory conditions

As the change angle increased, participants’ performance on the change detection task improved (see Fig. 2B). The significant main effect of change angle supported this observation (average *K* were 0.93 ± 0.09, 1.98 ± 0.59, and 2.71 ± 0.42 items) for the 15°, 30°, and 60° conditions, respectively; *F*(2,44) = 326.140, *p* < .001, η_*p*_^*2*^ = 0.940). Participants’ memory performance was better in the all-same condition than in the partial-same condition and the all-different condition, which was supported by the significant main effect of memory array condition on *K*, *F*(2,44) = 187.060, *p* < .001, η_*p*_^*2*^ = 0.900. We also found an interaction between the memory array and change angle, *F*(4,88) = 9.700, *p* < .001, η_*p*_^*2*^ = 0.310.

Planned pairwise comparisons revealed that when the change angle was 15°, the *K* values were significantly lower for the all-different condition than for the all-same condition, *t*(22) = 8.690, *p* < .001, *Cohen’s d* = 1.810, *BF*_*10*_ > 1000. Additionally, *K* was significantly higher in the all-same condition compared to the partial-same condition, *t*(22) = 5.810, *p* < .001, *Cohen’s d* = 1.210, *BF*_*10*_ > 1000. The partial-same condition also yielded significantly higher K than the all-different condition, *t*(22) = 2.250, *p* = .035, *Cohen’s d* = 0.470, *BF*_*10*_ = 1.770. When the change angle was 30°, the *K* values were significantly lower for the all-different condition than for the all-same condition, *t*(22) = 15.290, *p* < .001, *Cohen’s d* = 3.190, *BF*_*10*_ > 1000. Additionally, *K* was significantly higher in the all-same condition compared to the partial-same condition, *t*(22) = 8.990, *p* < .001, *Cohen’s d* = 1.880, *BF*_*10*_ > 1000. The partial-same condition also yielded significantly higher K than the all-different condition, *t*(22) = 5.150, *p* < .001, *Cohen’s d* = 1.070, *BF*_*10*_ = 626.35. When the change angle was 60°, the *K* values were significantly lower for the all-different condition than for the all-same condition, *t*(22) = 13.210, *p* < .001, *Cohen’s d* = 2.760, *BF*_*10*_ > 1000. Additionally, *K* was significantly higher in the all-same condition compared to the partial-same condition, *t*(22) = 10.970, *p* < .001, *Cohen’s d* = 2.290, *BF*_*10*_ > 1000. The partial-same condition also yielded significantly higher K than the all-different condition, *t*(22) = 5.850, *p* < .001, *Cohen’s d* = 1.220, BF_10_ > 1000. These findings consistently indicate superior memory performance in the all-same condition compared to the partial-same condition, and the all-different condition consistently yielded the lowest memory performance across all tested angles.

### ERP results

**Fig. 3:**
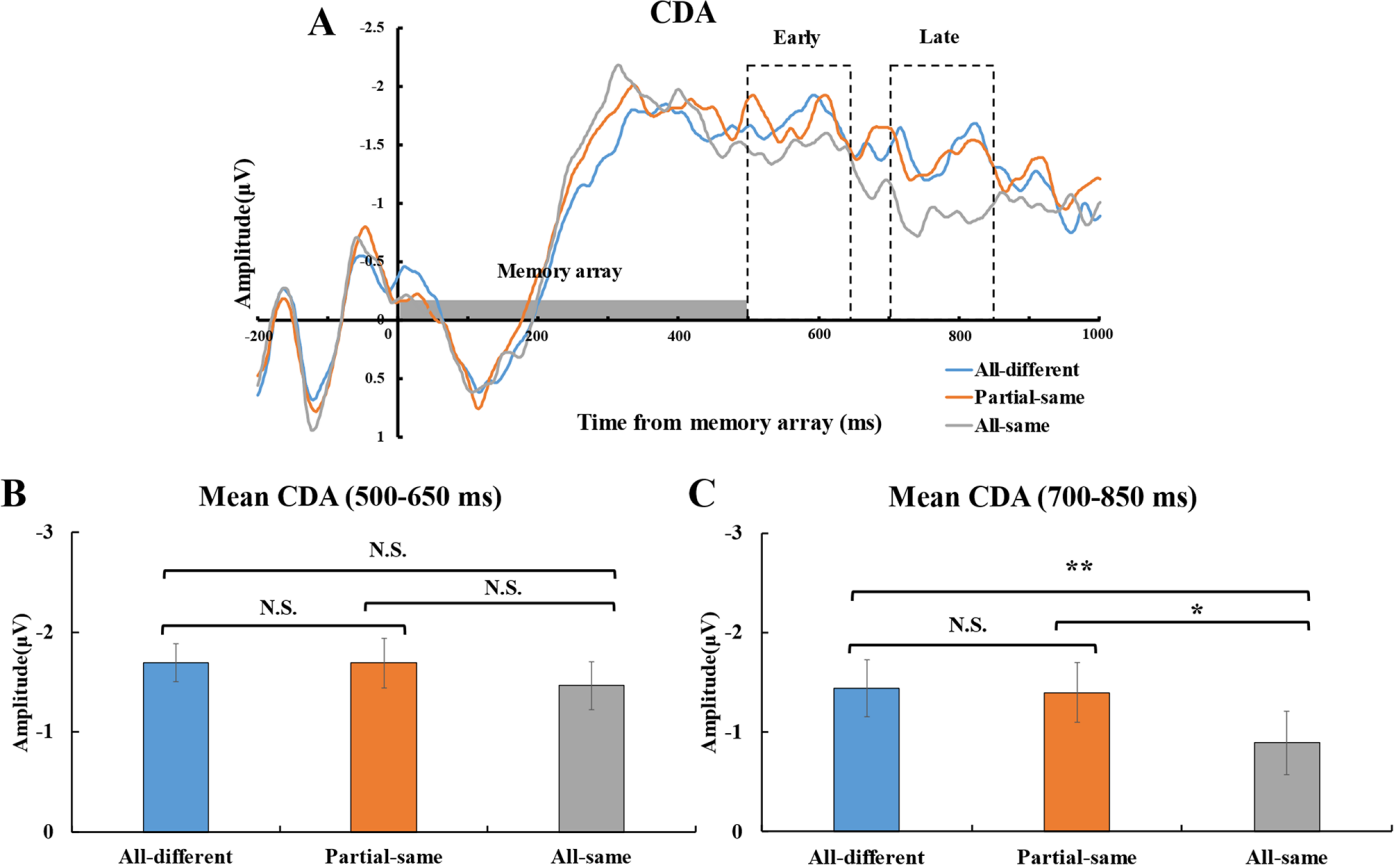
ERP results across memory conditions. (**A**) Grand-averaged waveforms (averaged across PO7/8 and P7/8 electrodes) for the three memory array conditions: all-different (blue), partial-same (orange), and all-same (gray). The waveforms reflect lateralized activity (contralateral minus ipsilateral) and are time-locked to the onset of the memory array (time zero on the x-axis). The shaded gray area indicates the duration of memory array presentation. Dashed rectangles highlight the predefined time windows for the early and late CDA components. (**B**) Mean CDA amplitudes during the early time window (500–650 ms) for each memory condition (all-different condition, partial-same condition, and all-same condition). (**C**) Mean CDA amplitudes during the late time window (700–850 ms) for each memory condition. Error bars indicate SE. ** = *p* < .01, * = *p* < .05, N.S. = *p* > .05.

The CDA amplitudes for each condition are depicted in Fig. 3. The two-way repeated measures ANOVA revealed a significant interaction between the time window, memory condition, *F*(2, 44) = 4.390, *p* = .018, η_*p*_^*2*^ = 0.170, a significant main effect of the time window, *F*(1, 22) = 4.380, *p* = .048, *η*_*p*_^*2*^ = 0.166, and a significant main effect of the memory condition, *F*(2, 44) = 4.570, *p* = .016, *η*_*p*_^*2*^ = 0.172.

### Early CDA result (500-650ms)

The averaged difference early CDA amplitudes for all-same condition, partial-same condition, and all-different condition are presented in Fig. 3B. The ANOVA revealed no significant main effect of the memory condition, *F*(2,44) = 1.920, *p* = .159, η_*p*_^*2*^ = 0.080.

Planned pairwise comparisons revealed that the early CDA amplitudes were no difference between the all-different condition (-1.70 ± 0.19 *µV*) and the all-same condition (-1.47 ± 0.24 *µV*), *t*(22) = 1.640, *p* = .116, *Cohen’s d* = 0.340, BF_10_ = 0.694. Additionally, the early CDA amplitudes showed no significant difference between the partial-same condition (-1.69 ± 0.25 *µV*) and the all-same condition, *t*(22) = 1.800, *p* = .086, *Cohen’s d* = 0.380, BF_10_ = 0.867. As well as, no significant differences were observed between the partial-same condition and the all-different condition, *t*(22) = 0.030, *p* = .976, *Cohen’s d* = 0.010, *BF*_*10*_ = 0.219. The results of early CDA amplitudes suggest participants did not reduce VWM resource consumption when the identical orientation were present during the early phase of VWM maintenance.

### Late CDA result (700–850 ms)

The averaged difference late CDA amplitudes for all-same condition, partial-same condition, and all-different condition are presented in Fig. 3C. The ANOVA revealed a significant main effect of the memory condition, *F*(2, 44) = 6.080, *p* = .005, η_*p*_^*2*^ = 0.217.

Planned pairwise comparisons revealed that the late CDA amplitudes were significantly larger for the all-different condition (-1.69 ± 0.25 *µV*) than the all-same condition (-0.89 ± 0.32 *µV*), *t*(22) = 2.810, *p* = .010, *Cohen’s d* = 0.590, BF_10_ = 4.860. Additionally, the CDA amplitudes was significantly higher in the partial-same condition (-1.44 ± 0.29 *µV*) compared to the all-same condition, *t*(22) = 2.680, *p* = .014, *Cohen’s d* = 0.560, *BF*_*10*_ = 3.783. However, no significant differences were observed between the partial-same condition and the all-different condition, *t* (22) = 0.330, *p* = .744, *Cohen’s d* = 0.070, *BF*_*10*_ = 0.230. The results of late CDA amplitudes suggest that participants only reduce VWM resource consumption when the all orientation were identical during the late phase of VWM maintenance.

### Exploratory analyses

Inspection of the grand-averaged lateralized waveforms revealed the presence of early perceptual ERP components even before the onset of the memory array. This is likely due to the presentation of the arrow cue 300 ms before the memory array. Because the cue-to-array interval was fixed, these early ERP components can be attributed to the processing of the arrow cue. However, based solely on the CDA analyses, we could not definitively determine the source of these early components. Therefore, we conducted additional exploratory analyses focusing on ERP components time-locked to the onset of the arrow cue. Epochs were re-segmented around arrow cue onset, using a 200 ms pre-cue baseline. We analyzed contralateral, ipsilateral, and lateralized (contralateral minus ipsilateral) activity.

These analyses revealed no significant ERP components prior to the arrow cue onset. Following the arrow cue onset, we observed several ERP components in both contralateral and ipsilateral waveforms, including a P1 (50–110 ms), N1 (110–200 ms), and P2 (210–300 ms). In the lateralized difference waveforms, an N2pc component was observed from approximately 200–290 ms after the arrow cue onset. These findings can explain the presence of early ERP components in the baseline period of the CDA grand average waveforms. Further details of the exploratory analyses related to the ERP components elicited by the arrow cue are provided in the Supplementary Materials.

## Discussion

The goal of this study was to examine how storing identical orientation stimuli impacts VWM processes. To this end, we systematically manipulated the configuration of memory arrays containing four orientation stimuli. This manipulation resulted in three distinct experimental conditions: an all-different condition (four orientation stimuli, each with a different orientation), a partial-same condition (four orientation stimuli, consisting of two pairs with identical orientations), and an all-same condition (four orientation stimuli, all with the same orientation). These conditions corresponded to “effective set sizes” of four, two, and one unique orientation features, respectively.

The behavioral results revealed a progressive improvement in the VWM performance as the number of identical orientations increased (i.e., as the effective set size decreased). This finding indicates that participants benefited from enhanced VWM performance in both the all-same and partial-same conditions when storing multiple identical objects. These results are consistent with previous behavioral studies showing improved VWM performance when storing identical items^45,50^. Additionally, similar patterns were observed across change angles of 15°, 30°, and 60°, suggesting that while change angles or task difficulty may influence overall VWM performance across different memory conditions, they do not alter the performance advantage gained from storing identical objects.

More importantly, we sought to identify the specific conditions and temporal windows in which this effect emerges. We examined differences in CDA amplitudes across different memory conditions. The CDA results were partially asynchronous with the behavioral results. In the early time window, there were no significant differences in CDA amplitudes across the all-same, partial-same, and all-different conditions. However, in the late time window, the CDA amplitude for the all-same condition was significantly lower than those of the partial-same and all-different conditions. This finding suggests that reductions in VWM resource consumption occur exclusively during the late maintenance phase and only when all orientations are identical. This also indicates that even under all-same conditions, participants initially store all identical orientations in VWM independently, but subsequently reduce VWM resource consumption during the late maintenance phase. In contrast, CDA amplitudes did not differ between the partial-same and all-different conditions in either the early or late time windows. This indicates that the consolidation of partially identical orientation pairs does not facilitate the allocation of VWM resources, and these items are presumably maintained as four independent items, similar to the all-different condition.

The differences in CDA patterns observed across the early and late maintenance phases likely reflect distinct underlying VWM processes. Specifically, participants did not exhibit reduced VWM resource allocation during the early maintenance phase but demonstrated a significant reduction under the all-same condition during the late maintenance phase. Specifically, the absence of CDA reduction during early maintenance, paired with the significant reduction during late maintenance in the all-same condition, suggests a late-selection strategy by the VWM system rather than stimulus-driven, preattentive chunking. This finding argues against the idea that identical stimuli automatically form prepackaged units prior to or during the early consolidation phase. Rather, in the early maintenance phase immediately following stimulus offset, VWM appears primarily engaged in consolidating each individual perceptual representation separately. In the late maintenance phase, however, when stimuli are identical, VWM adaptively identifies the common features across stored VWM representations, resulting in one unified, shared representation. Such a late-selection consolidation effectively reduces cognitive load, facilitating simultaneous maintenance of multiple identical items.

Notably, while our evidence supports the notion that the benefit of storing identical orientations emerges only during the late maintenance phase, this late-selection mechanism might still operate as a spontaneous process, independent of voluntary control. For instance, previous studies have demonstrated that even with discrete items within the memory array, visual information connections can facilitate automatic binding, thereby improving VWM performance^1,28,45,89–92^. However, such automatic binding processes within VWM may require time to develop during the maintenance period, resulting in a reduction in CDA amplitude only during the late maintenance phase as intra-item features or separate items are integrated into VWM^66,93^. This further suggests that while perceptual processing of identical objects in visual perception appears to automatically enhance performance^37^, the same benefit from perceptual process does not directly translate into VWM during the early consolidation phase. Despite the substantial overlap between perceptual and VWM processes^41^, the strong benefits of perceptual organization observed in visual perception are not immediately reflected in VWM. Instead, under specific conditions (e.g., when all stimuli are identical), individuals can gain additional benefits from identical object organization during the late maintenance phase of VWM. This observation helps to explain why previous studies in visual perception reported performance benefits under both all-same and partial-same conditions^38^, whereas our CDA findings only observed reduced VWM resource consumption in the all-same condition.

Interestingly, this reduction in the neurally active load signal (i.e., CDA amplitude) was observed exclusively in the all-same condition, but not in the partial-same condition. The results under the partial-same condition align with findings from Shen, et al.^67^. A plausible explanation for the lack of effect in the partial-same condition is that it may depend on the immediate availability of shared feature categorization. That is, in the all-same condition, extracting a single shared feature during the late-selection is straightforward, allowing for a unified representation of all items. Conversely, in the partial-same condition, categorizing two pairs of identical orientations and selectively reallocating internal attention to the two regions of the memory array would be more challenging, especially since the locations of partially identical items were randomized across trials. Because the identical object effect occurs during the maintenance phase rather than before the early maintenance phase, the complexity of the partial-same condition likely hinders effective late selection, preventing the VWM system from efficiently representing two distinct orientations from the four items. As a result, the cognitive load is not alleviated in the partial-same condition as it is in the all-same condition.

Despite the absence of reduced CDA activity in the partial-same condition, behavioral results still showed an advantage for the partial-same condition over the all-different condition. Our CDA findings in the partial-same condition suggest that participants generally maintain all individual item representations, even when some items are identical. Consequently, the VWM resource reduction effect was observed only when all items were identical, as in the all-same condition. This indicates that performance improvements due to identical items are not solely contingent on reduced VWM resource deployment. Instead, the behavioral benefit observed in the partial-same condition may stem from other factors, such as the relative ease of item individuation, reduced inter-item interference during maintenance, or more efficient decision-making processes during change detection tasks^59,92,94^. Thus, the results of the current study provide tentative evidence supporting the notion that the effects of perceptual organization, even if absent during VWM maintenance phases, can influence VWM processes during retrieval and comparison phases. Future research could further explore these mechanisms by employing paradigms similar to ours but incorporating methods beyond CDA. For instance, decoding “effective set sizes” through multivariate analyses of raw voltage signals^95–98^ may provide deeper insights into the cognitive mechanisms underlying the observed behavioral benefits. Taken together, our results support the “Complete Identicality Benefit Effect” hypothesis, demonstrating that the facilitative effect of identical orientation stimuli on the number of items storing in VWM is contingent upon all items being identical. These findings align with the ERP finding by Gao, et al.^65^ and Shen, et al.^67^, but contrast with Peterson, et al.^66^, who found identical colors could be integrated even under a partial-same condition. However, it is important to note that findings derived from orientation stimuli may not directly generalize to color stimuli. Thus, our results should not be interpreted as a direct challenge to Peterson, et al.^66^. It is possible that colors naturally facilitate participants’ ability to quickly identify and integrate identical features, even when identical colors are interspersed with other colors. In contrast, for orientation stimuli, efficient integration may depend on specific spatial configurations, adding complexity to the partial-same condition. Our analysis of CDA across different time windows also supports the conclusion that orientational stimuli are more challenging to integrate. There were no differences in the early window among the three conditions, but a significant decrease in the CDA amplitude for the all-same condition in the late window, compared to the other two conditions. This suggests that even when all stimuli within the visual field are of the same orientation, participants require a brief period for discernment and integration of the identical orientational stimuli. Overall, the effect observed under the all-same condition arises because the visual system can efficiently integrate identical stimuli into a single representation, thereby reducing VWM resource consumption. In contrast, the partial-same condition does not elicit this benefit because the presence of both identical and non-identical items complicates the integration process. This results in participants defaulting to maintaining all items in VWM, which prevents resource consumption reduction.

Another interpretation of the results is that the all-same condition requires no active processes in memory since in that condition there is no competition of resources and no distracting information. In all-different and partial-same conditions, different orientations either compete with or distract each other, leading to an increase in cognitive load. Since three memory array conditions all have four memory items, the results suggest that CDA is only sensitive to active memory processing, but not to the number of information/orientations in memory array. This implies that CDA is a sensitive measure of whether information is actively being processed in VWM. Previous research has also found that the CDA amplitude is not solely related to the number of items in VWM (memory load) but is influenced by the relevance and prioritization of items^99^. This further supports the idea that the CDA amplitude is more closely tied to the cognitive processes involved in maintaining and manipulating information in VWM, rather than the mere quantity of items held in memory. Therefore, the absence of a difference in CDA amplitude between the all-different and partial-same conditions suggests that the active processing of the orientations in memory is similar in both conditions, despite the presence of more orientations in the all-different condition. This is consistent with our conclusion that strategic late-selection and consolidation are necessary for integrating identical orientations. Future research could further validate these interpretations by examining VWM-related neural activity using paradigms similar to ours and using advanced neuroimaging techniques such as fMRI^49,100,101^.

It is worth noting that recent behavioral studies using orientation stimuli have shown that when participants memorize two pairs of identical orientations (similar to our partial-same condition), VWM precision is superior when each pair of identical orientations is adjacent. However, when identical orientations are not adjacent, VWM performance does not significantly differ from that in the all-different condition^50^. These findings suggest that the VWM performance benefit derived from identical objects may depend on their spatial proximity, with adjacency facilitating the benefit. This perspective is also supported by the study by Peterson and Berryhill^45^, which used colors as stimuli. They controlled the spatial adjacency of identical colors in their partial-same condition and found that the benefit of identical stimuli for VWM performance emerged only when the identical items were completely adjacent. Together, these results indicate that spatial proximity amplifies the benefits of identical objects in VWM. It is important to note that in previous behavioral studies, memory arrays were bilaterally presented, requiring participants to memorize stimuli across both visual hemifields. Under such conditions, non-adjacent identical objects are often distributed across contralateral visual fields, potentially hindering the benefits of identical objects. Research has shown that resource allocation for VWM is partially independent across the two visual hemifields. For example, studies have demonstrated better VWM performance when items are distributed across both hemifields compared to when they are restricted to a single hemifield, a phenomenon termed the bilateral field advantage^102,103^. This advantage likely arises from increased attentional resource allocation when items are presented across both hemifields^104^. Additionally, greater negative activity is often observed in the contralateral parietal-occipital cortex when stimuli are confined to one hemifield, reflecting the role of each hemisphere in supporting contralateral VWM representations^57,105,106^. Thus, previous studies may have failed to observe VWM benefits for non-adjacent identical objects due to the disruptive influence of inter-hemifield stimulus distribution. In our study, however, identical orientations, whether adjacent or non-adjacent, were always presented within the same visual hemifield, avoiding potential complications arising from inter-hemifield distribution. To further investigate the role of spatial proximity in the storage of identical stimuli, we conducted an additional analysis of our behavioral data, focusing on three sub-conditions within the partial-same condition: (1) separated-same condition, in which each pair of identical orientations was separated by an intervening item (no identical orientations were adjacent); (2) central-peripheral-same condition, in which one pair of identical orientations was positioned centrally while the other pair was located peripherally (one pair of identical orientations was adjacent); and (3) adjacent-same condition, in which each pair of identical orientations was adjacent (two pairs of identical orientations were adjacent). Due to the randomized distribution of stimuli, the separated-same condition accounted for 50% of trials, while the central-peripheral-same and adjacent-same conditions each accounted for 25% of trials within the partial-same condition. Our results revealed that VWM performance in all three sub-conditions was better than in the all-different condition, indicating that identical objects confer benefits even when they are not adjacent, provided that they are presented within the same visual hemifield. Notably, VWM performance was highest in the adjacent-same condition and lowest in the separated-same condition, suggesting that spatial proximity and the presence of identical objects contribute additively to VWM performance (more detailed analyses and discussions of these findings can be found in the Supplementary Materials). Interestingly, Peterson, et al.^66^ demonstrated that in partial-same conditions, the adjacency of identical objects did not influence CDA amplitude. Together, these results suggest that while spatial proximity may affect behavioral performance, it does not influence the VWM resources occupied by identical objects, as indexed by CDA amplitude.

It is also worth noting that our CDA results caution against assuming a linear relationship between CDA amplitude and effective set size when memory arrays include multiple identical stimuli. Specifically, our data suggest that the neural signatures of VWM load do not monotonically scale according to the effective set sizes manipulated in our experiment (set size four in all-different condition, set size two in partial-same condition, and set size one in all-same condition). Therefore, in VWM tasks featuring multiple identical items, it may be inappropriate to assume, without explicit empirical validation, that the cognitive load associated with storing several identical stimuli equates straightforwardly to that of storing a single stimulus.

An important consideration is whether the presence of adjacent identical objects in the partial-same condition leads to a reduction in VWM resource consumption, as reflected in CDA amplitude. To investigate this, one could compare the CDA amplitudes across the separated-same, central-peripheral-same, and adjacent-same sub-conditions with that of the all-different condition. Unfortunately, calculating reliable CDA amplitudes requires a sufficient number of trials per condition^107^. In our study, the limited number of trials in each sub-condition made it impractical to conduct such an analysis. However, we can draw inferences from existing evidence. Within the partial-same condition, trials involving adjacent identical objects (i.e., central-peripheral-same and adjacent-same sub-conditions) accounted for 50% of the total trials. If adjacent identical objects contributed to reduced VWM resource consumption, as suggested by lower CDA amplitudes, this trend would be expected to influence the overall CDA amplitude for the partial-same condition. Yet, our findings did not reveal any trend toward a reduction in CDA amplitude for the partial-same condition compared to the all-different condition in either the early or late time windows. These results suggest that the adjacency of identical objects in the partial-same condition does not lead to measurable reductions in VWM resource consumption. These results indicate that our study did not find evidence to support the notion that the adjacency of identical objects in the partial-same condition leads to reductions in VWM resource consumption.

In summary, this study examined how the presence of identical orientation stimuli influences VWM resource allocation, shedding light on the temporal dynamics and cognitive mechanisms underlying the benefits of identical objects in VWM. Using ERP techniques, we found a significant reduction in VWM resource consumption exclusively when all stimuli were identical, and only during the late maintenance phase. This finding suggests that the benefits of identical objects in VWM do not arise during the early maintenance phase but rather during a late-selection process, where shared features of identical stimuli are consolidated into unified representations. In contrast, the presence of partially identical objects did not alleviate VWM resource consumption. This discrepancy underscores the complexity of processing partially identical stimuli, which likely hinders the efficient integration of these items into fewer memory representations. Our findings support the hypothesis that the facilitative effect of identical objects on VWM resource consumption depends on the complete uniformity of stimuli and is driven by a strategic late-selection mechanism. These results offer valuable insights into the temporal dynamics of VWM processes and emphasize the distinct roles of encoding, maintenance, and retrieval phases in leveraging perceptual organization to optimize cognitive performance. Future research should build on these findings by using advanced neuroimaging techniques and multivariate analyses to further investigate how identical objects affect VWM across diverse task conditions and feature dimensions.

## Electronic supplementary material

Below is the link to the electronic supplementary material.

Supplementary Material 1

## Data Availability

The datasets generated during and/or analysed during the current study are available in the Open Science Framework at https://osf.io/j6yse/.
