# Supplementary material for: All identical objects reduce memory load at the late maintenance stage in working memory
Source: Sci Rep. 2025 May 14;15:16700. doi: 10.1038/s41598-025-00433-4 (PMC12078691; doi:10.1038/s41598-025-00433-4)
Supplement: Supplementary file 1 — Supplementary Material 1 [file 41598_2025_433_MOESM1_ESM.pdf]

Supplememtary Materials

All Identical Objects Reduce Memory Load at the Late Maintenance Stage in Working Memory

Lijing Guo [1, 2], Hyung-Bum Park [3], Guofang Ren [1], Penglan Liu [2], Ruyi Liu [2,4], Dan Nie [2], Chaoxiong Ye [1,2 *]

[1] School of Education, Anyang Normal University, Anyang, China
[2] Department of Psychology, University of Jyvaskyla, Jyvaskyla, Finland
[3] Institute for Mind and Biology, University of Chicago, USA
[4] Institute of Brain and Psychological Sciences, Sichuan Normal University, Chengdu, China

*** Correspondence to:**
Chaoxiong Ye, PhD
School of Education
Anyang Normal University, Anyang, China
Email: cxye1988@163.com

1 Pilot experiment

To better validate the effectiveness of the experimental task control, we conducted a behavioral pilot experiment. Similar to the main experiment, the pilot included three conditions: all-same (i.e., four identical orientations), partial-same (i.e., two pairs of identical orientations), and all-different (i.e., four unique orientations). Additionally, to prevent participants from forming a fixed expectation regarding the change magnitude of the probe items, we introduced varied change angles. We hypothesize that, under the same change angle, participants will perform better in the all-same condition compared to the partial-same condition, and performance in the partial-same condition will be superior to the all-different condition. Furthermore, when the change angle is smaller, participants are expected to find it more challenging to detect the change, resulting in poorer performance.

Participants

A group of 24 participants from Sichuan Normal University took part in the study and received monetary compensation. The inclusion criteria were 18 years of age or older, self-reported normal color vision, and normal or corrected-to-normal visual acuity. Exclusion criteria included a history of psychiatric disorders, use of nervous system acting drugs, and previous participation in working memory experiments. One participant was excluded due to a correct response rate below 50%. As a result, 23 participants (22 females and 1 male, with an average age of 19.913 years; age range 18-22 years, SD ±1.505) were included in the final analysis. This study was approved by the Ethics Committee of the Institute of Brain and Psychological Sciences, Sichuan Normal University.

Stimuli

The experimental procedure was implemented using E-Prime software. Stimuli were presented on a 23.8-inch LCD monitor with a resolution of 1280 × 768 pixels and a refresh rate of 60 Hz. The screen background was black (RGB: 0, 0, 0). Participants were seated approximately 60 cm from the screen, ensuring a consistent viewing distance. A white fixation cross (RGB: 255, 255, 255) was displayed at the center of the screen throughout the experiment to maintain focus. The memory and probe stimuli were composed of white bars (RGB: 255, 255, 255). In the memory array, eight bars were presented in a circular arrangement around the central fixation cross, with a radius of 5 visual angle (dva). The bars were symmetrically distributed between the left and right hemifields relative to the fixation point. Each bar measured 1.4 dva × 0.2 dva in size, with an inter-bar spacing of 2.9 dva and a distance of 3.3 dva from the fixation point. In each trial, the possible orientations of the bars in the memory array were randomly chosen from 180 possible angles, with a minimum

angular separation of 45° between any two bars to ensure distinct orientations. In the probe array, one bar was presented at a randomly selected location in each hemifield, corresponding to the position of a bar from the memory array. If the probe orientation differed, the angular deviation was randomly selected from four possible values: 15°, 30°, 45°, or 60°. These angular changes were applied with equal probability across trials to systematically vary the difficulty of the memory-matching task.

Procedure

The experimental procedure is depicted in SFigure 1. A central fixation cross remained visible throughout the experiment to help participants maintain focus. Each trial began with a cue phase lasting 200 ms, during which an arrow appeared above the fixation cross, pointing either to the left or right with equal probability. The arrow cued participants to remember the orientations of the bars presented in the corresponding hemifield while ignoring the bars in the opposite hemifield. Following the cue phase, a variable blank interval of 100–200 ms was presented to allow participants to process the cue and prepare for the memory array. During this interval, the fixation cross remained visible. Next, a memory array was presented for 500 ms, containing eight bars symmetrically divided between the two hemifields (four bars per side). The memory array was designed with three conditions: (1) the all-same condition, where all bars in each hemifield shared the same orientation; (2) the partial-same condition, where each hemifield contained two pairs of bars with identical orientations; and (3) the all-different condition, where all bars in each hemifield had unique orientations. Participants were instructed to memorize the orientations of the bars in the cued hemifield. Following the memory array, a maintenance phase lasted for 1000 ms, during which the screen was blank, and only the fixation cross was displayed. Participants were required to retain the memorized orientations during this phase. In the subsequent probe phase, a single bar appeared at a random location in each hemifield, corresponding to the position of a bar from the memory array. Each probe bar either matched the orientation of the corresponding bar in the memory array (50% chance) or differed by 15°, 30°, 45° or 60°, with equal probability for each change angle. Participants were instructed to indicate whether the probe orientation matched their memory of the original bar by pressing the "F" key for a match and the "J" key for a mismatch. The trial ended after the participant’s response or after 2000 ms if no response was provided. Finally, a feedback phase lasted for 500 ms, during which "correct" or "incorrect" was displayed based on the participant’s response. An inter-trial interval, randomized between 900 and 1100 ms, was presented before the onset of the next trial.

Prior to the pilot experiment, participants completed 24 practice trials to familiarize themselves with the task structure and requirements. The pilot experiment consisted of 768 trials, evenly distributed across the three memory conditions (256 trials per condition). Trials from all conditions were presented in a randomized order. The entire session lasted approximately one hour, including 11 scheduled breaks to reduce fatigue and maintain high data quality.

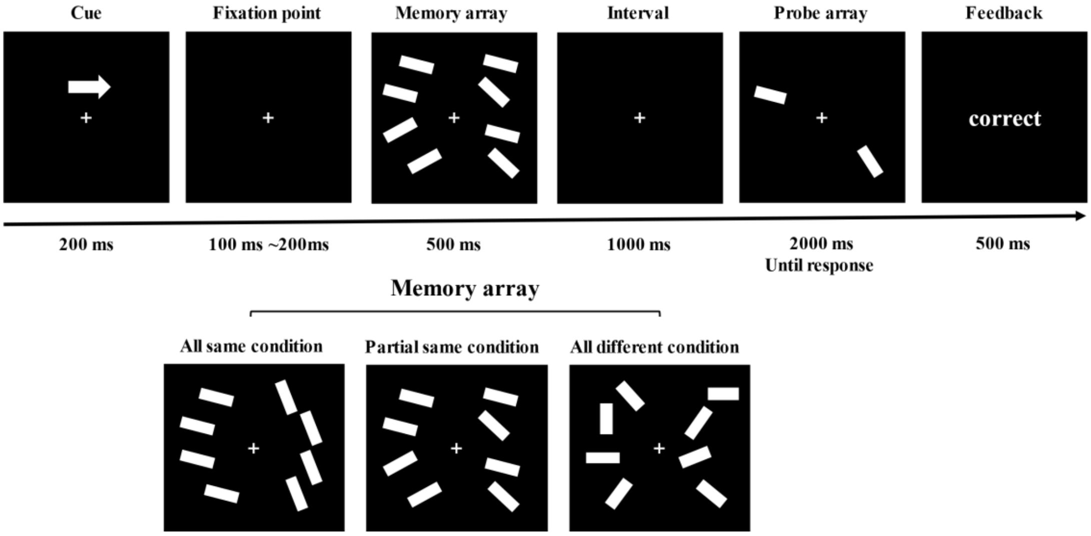

SFigure 1: (A) Flowchart of the experimental task. (B) Three conditions of the memory array: all-same condition; partial-same condition; all-different condition.

Data analysis

We used *Cowan's K* to quantify VWM performance [1]. Cowan's K was calculated with the formula: $K = N \times (H-F)$, where N is the set size of memory array (four in our experiment), H is the hit rate (the proportion of correct responses when a change was present), and F is the false alarm rate (the proportion of incorrect responses when no change was present). Higher K values indicate better memory performance. First, we ran a one-way repeated-measures ANOVA to examine the effect of memory condition (all-same, partial-same, all-different) on K values. Planned pairwise comparisons were performed using two-tailed paired t-tests to compare differences between each pair of conditions (all-same vs. partial-same, all-same vs. all-different, and partial-same vs. all-different). Next, to further investigate the effect of change angle on different memory conditions, we conducted a two-way repeated-measures ANOVA with memory condition (all-same, partial-same, all-different) and change angle (15°, 30°, 45°, 60°) as within-subject factors on K values. Planned pairwise comparisons for each change angle were conducted with two-tailed paired t-tests to compare differences among the three memory conditions. The effect size for ANOVA was estimated using the partial eta-squared (η_p^2) value. JASP (version 0.19) was used to provide *Cohen's d*, estimating the effect size for the t-tests, and Bayes factors [2], showing whether the t-test results supported the alternative hypothesis, thereby providing an odds ratio for the alternative/null hypotheses (values < .3 provide evidence for the null hypothesis and values >3 provide evidence for the alternative hypothesis).

Result

The mean *K* values for each memory condition (all-same condition vs. partial-same condition vs. all-different condition) is presented in SFigure 2A. The analysis of variance (ANOVA) revealed a significant main effect of the *K* (mean *K* for the all-same condition, partial-same condition, and all-different condition: 2.632 ± 0.145, 1.352 ± 0.112, 0.970 ± 0.094 items, respectively), $F(2, 44) = 239.131$, $p < .001$, $\eta_p^2 = 0.916$.

Planned pairwise comparisons revealed that the *K* were significantly lower for the all-different condition than for the all-same condition, $t(22) = 16.611$, $p < .001$, *Cohen's d* = 3.464, $BF_{10} > 1000$. Additionally, the *K* showed a significant difference between the partial-same condition and the all-same condition, $t(22) = 16.812$, $p < .001$, *Cohen's d* = 3.506, $BF_{10} > 1000$, and significant differences were observed between the partial-same condition and the all-different condition, $t(22) = 6.745$, $p < .001$, *Cohen's d* = 1.407, $BF_{10} > 1000$. These *K* results suggest that the performance of VWM improved with the number of identical orientations increases.

As the change angle increased, participants' performance on the change detection task improved (see SFigure 2B). The significant main effect of change angle supported this observation for the 15°, 30°, 45° and 60° conditions, respectively; $F(3,66) = 126.671$, $p < .001$, $\eta_p^2 = 0.852$. Participants' memory performance was better in the all-same condition than in the partial-same condition and the all-different condition, which was supported by the significant main effect of memory array condition on *K*, $F(2,44) = 239.131$, $p < .001$, $\eta_p^2 = 0.916$. We also found an interaction between the memory array and change angle, $F(6,132) = 6.757$, $p < .001$, $\eta_p^2 = 0.235$.

Planned pairwise comparisons revealed that when the change angle was 15°, the *K* values were significantly lower for the all-different condition than for the all-same condition, $t(22) = 5.725$, $p < .001$, *Cohen's d* = 1.194, $BF_{10} > 1000$. Additionally, the *K* showed a significant difference between the partial-same condition and the all-same condition, $t(22) = 7.165$, $p < .001$, *Cohen's d* = 1.494, $BF_{10} > 1000$, but no significant differences were observed between the partial-same condition and the all-different condition, $t(22) = 0.360$, $p = .715$, *Cohen's d* = 0.233, $BF_{10} = 0.221$. When the change angle was 30°, the *K* values were significantly lower for the all-different condition than for the all-same condition, $t(22) = 12.469$, $p < .001$, *Cohen's d* = 2.600, $BF_{10} > 1000$. Additionally, the *K* showed a significant difference between the partial-same condition and the all-same condition, $t(22) = 10.704$, $p < .001$, *Cohen's d* = 2.251, $BF_{10} > 1000$, and significant differences were observed between the partial-same condition and the all-different condition, $t(22) = 2.954$, $p = .007$, *Cohen's d* = 0.616, $BF_{10} = 6.375$. When the change angle was 45°, the *K* values were significantly lower for the all-different condition than for the all-same condition, $t(22) = 12.920$, $p < .001$, *Cohen's d* = 2.694, $BF_{10} > 1000$. Additionally, the *K* showed a significant difference between the partial-same condition and the all-same condition, $t(22) = 9.667$, $p < .001$, *Cohen's d* = 2.016, $BF_{10} > 1000$, and significant differences were observed between

the partial-same condition and the all-different condition, $t(22) = 4.955$, $p < .001$, *Cohen's d* = 1.033, $BF_{10} = 439.123$. When the change angle was 60°, the *K* values were significantly lower for the all-different condition than for the all-same condition, $t(22) = 10.711$, $p < .001$, *Cohen's d* = 2.233, $BF_{10} > 1000$. Additionally, the *K* showed a significant difference between the partial-same condition and the all-same condition, $t(22) = 8.277$, $p < .001$, *Cohen's d* = 1.726, $BF_{10} > 1000$, and significant differences were observed between the partial-same condition and the all-different condition, $t(22) = 3.027$, $p = .006$, *Cohen's d* = 0.631, $BF_{10} = 7.355$.

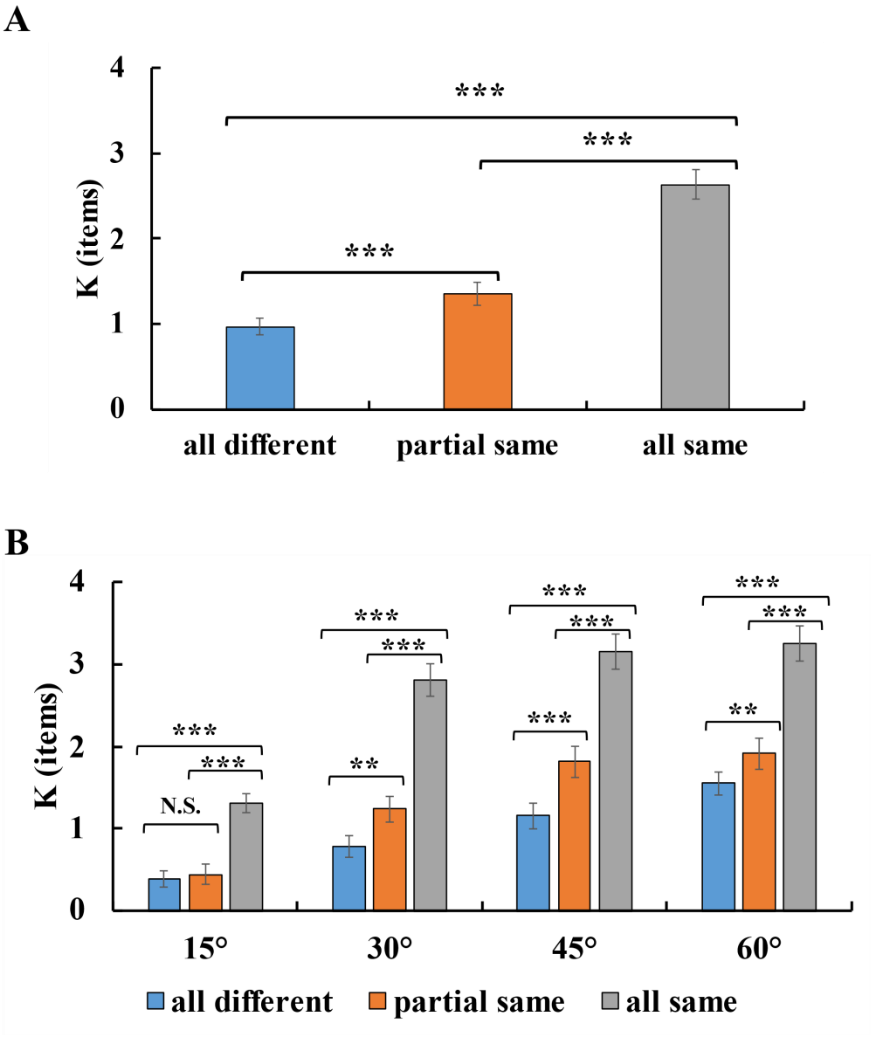

SFigure 2: Cowan's K results for each condition. (A): Mean and standard error of the mean for the K under different memory array conditions (all-same condition, partial-same condition, and all-different condition). (B) K results for the three memory arrays under four different conditions of angle change. Error bars indicate SE. N.S. = no significant difference, **= $p < .01$ *** = $p < .001$.

Discussion

Consistent with our initial expectations, participants' VWM performance improved both as the number of identical objects increased and as the change angle became larger. These findings confirm the effectiveness of our manipulation of identical object quantity. Notably, we observed a significant interaction between memory condition and change angle. Specifically, when the change angle was 15°, there was no statistically significant difference between the all-different and partial-same conditions. However, at angles greater than 15°, the partial-same condition began to outperform the all-different condition. A plausible explanation is that participants derive relatively limited benefits from identical objects in the partial-same condition, and these benefits appear to be sensitive to the difficulty of the comparison phase in the probe array. This pattern indicates that the advantage of identical objects in the partial-same condition may primarily manifest during the retrieval and comparison phases of VWM. Confirming this possibility will require evidence from the ERP experiments discussed in the main study. In contrast, our results show that the all-same condition consistently provided a robust VWM advantage across all tested change angles, suggesting a more stable contribution from identical objects when all four orientations are identical. Overall, the pilot experiment demonstrates that the memory conditions we designed are effective in modulating VWM performance at a behavioral level.

Finally, because VWM performance under the 45° condition was closely aligned with the 60° condition, we omitted the 45° change angle in the main experiment to enhance the discriminability among the remaining angles. Consequently, the main experiment includes only 15°, 30°, and 60° change angles.

2 Exploratory analysis of ERP components elicited by the arrow cue

During the baseline period for the CDA component (-200 to 0 ms), we observed substantial fluctuations, which are relatively uncommon in previous studies. We propose that this pre-memory-array ERP activity was elicited by the arrow cue presented prior to the memory array. In our experiment, the arrow cue was presented for 200 ms and consistently appeared 300 ms before the onset of the memory array. As a result, the perceptual processing of the cue itself, along with the attentional shifts it elicited, may have elicited early ERP components. These effects were evident in the grand-averaged lateralized waveforms shown in the main text, which revealed the presence of perceptual ERP components even before the onset of the memory array.

However, based solely on the CDA analyses, we could not definitively determine the source of these early components. Therefore, we conducted additional exploratory analyses focusing on ERP components time-locked to the onset of the arrow cue. Epochs were re-segmented around arrow cue onset, using a 200 ms pre-cue baseline. We analyzed contralateral, ipsilateral, and lateralized (contralateral minus ipsilateral) activity.

Methods

To investigate the ERP components elicited by the arrow cue, we reanalyzed the EEG data with a focus on cue-evoked activity. Since the arrow cue was identical across the three memory conditions (all-same, partial-same, and all-different), we collapsed data across these conditions and only distinguished trials based on cue direction (left vs. right). Cue onset was defined as 0 ms, and a 200 ms pre-cue interval served as the baseline for correction. This analysis focused on EEG activity occurring in the interval between cue onset and memory array onset.

EEG data were processed offline using MATLAB (2019). EEG signals were segmented into 500 ms epochs, spanning from -200 ms to 300 ms relative to cue onset. A low-pass filter with a cutoff frequency of 30 Hz was applied. Baseline correction was performed by subtracting the mean amplitude of the 200 ms pre-cue interval (-200 to 0 ms). Trials containing horizontal eye movements, identified by electrooculogram (EOG) activity exceeding ±60 μV, were excluded. Additionally, trials with remaining artifacts exceeding ±80 μV in amplitude were rejected.

ERP components were analyzed at two pairs of posterior electrode sites (PO7/PO8 and P7/P8), where cue-related visual components are typically observed. Contralateral waveforms were computed by averaging the activity recorded at the left hemisphere electrodes when the cue was directed to the right side, and vice versa for right hemisphere electrodes when the cue was directed to the left. Ipsilateral waveforms were obtained by averaging the activity recorded at the left and right hemisphere sites when the cue was directed to the left and right sides, respectively.

The difference waveform was then derived by subtracting ipsilateral from contralateral activity, yielding three distinct waveforms: contralateral, ipsilateral, and difference waves. These waveforms provide a clear representation of cue-evoked neural activity.

Visual inspection of the averaged waveforms (SFigure 3) revealed distinct ERP components in response to cue onset, including a positive peak around 50–110 ms (P1), a negative peak around 110–200 ms (N1), a positive peak around 210– 300 ms (P2) and a sustained negativity around 200–290 ms (N2pc). These time windows align with previous findings on early visual processing and attentional selection (e.g., Hillyard and Anllo-Vento, 1998; Vogel, & Luck, 2000; Woodman & Luck, 1999).

To quantify these components, we employed the individual peak amplitude extraction method for P1, N1, and P2. Specifically, for each participant, we identified the largest positive peak for P1 within the 50–110 ms window, P2 within the 210–300 ms window, and the most negative peak for N1 within the 110–200 ms window in both contralateral and ipsilateral waveforms. For N2pc, since it reflects sustained lateralized attentional selection rather than a sharp peak (Luck & Hillyard, 1994), we computed the mean amplitude within the 200–290 ms time window from the difference waveform.

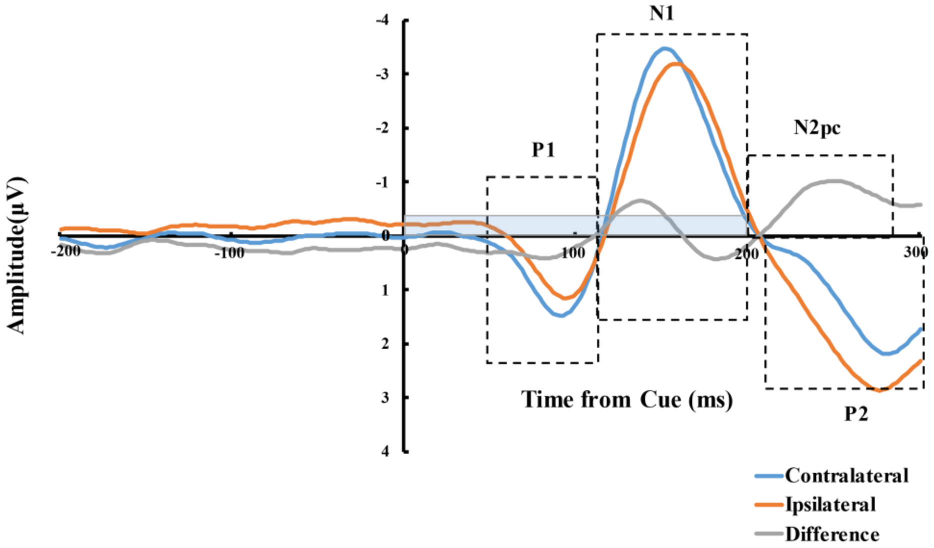

SFigure 3: ERP components evoked by the cue, shown as three distinct waveforms: difference wave (gray), contralateral wave (blue), and ipsilateral wave (orange). The waveforms are time-locked to cue onset (time zero on the y-axis). The shaded blue box indicates the cue presentation period. The time windows for the ERP components are as follows: P1 (50–110 ms), N1 (110–200 ms), P2 (210–300 ms), and N2pc (200–290 ms).

Statistical Analysis

To assess whether the cue reliably evoked these ERP components, we conducted one-sample t-tests against zero for the extracted amplitudes of P1, N1, P2, and N2pc. Specifically, we tested whether the peak amplitudes of P1, N2, and P2 (from contralateral and ipsilateral waveforms) were significantly different from zero, and

whether the mean amplitude of N2pc in the difference waveform (contralateral minus ipsilateral) during the 200–290 ms window was significantly below zero. Additionally, to examine whether cue-evoked activity differed across hemispheres, we performed a one-way repeated-measures ANOVA on P1, N1, and P2 amplitudes, with hemisphere (contralateral vs. ipsilateral) as the within-subject factor. A significant main effect would indicate lateralization in early visual processing.

Results

The cue elicited a significant P1 response. The peak amplitudes of P1 were significantly greater than zero for both contralateral waveform, $t(22) = 6.225$, $p < .001$, Cohen's d = 1.298, $BF_{10} > 1000$, and ipsilateral waveform, $t(22) = 5.825$, $p < .001$, *Cohen's d* = 1.215, $BF_{10} > 1000$, and the peak amplitudes of P2 were significantly greater than zero for both contralateral waveform, $t(22) = 8.318$, $p < .001$, *Cohen's d* = 1.734, $BF_{10} > 1000$, and ipsilateral waveform, $t(22) = 10.789$, $p < .001$, *Cohen's d* = 2.250, $BF_{10} > 1000$. Similarly, the cue evoked a significant N1 response, with peak amplitudes significantly below zero for contralateral waveform, $t(22) = 6.745$, $p < .001$, *Cohen's d* = 1.406, $BF_{10} > 1000$, and ipsilateral waveform, $t(22) = 6.513$, $p < .001$, *Cohen's d* = 1.358, $BF_{10} > 1000$. The cue also elicited a significant N2pc component. The mean amplitude of N2pc in the difference waveform during the 200–290 ms window was significantly below zero, $t(22) = 7.528$, $p < .001$, *Cohen's d* = 1.570, $BF_{10} > 1000$.

A one-way repeated-measures ANOVA on P1, N1, and P2 amplitudes revealed a significant main effect of hemisphere for P1, $F(1,22) = 17.935$, $p < .001$, $\eta_p^2 = 0.449$, suggesting lateralization in early visual processing. For N1, the main effect approached significance, $F(1,22) = 3.204$, $p = .087$, $\eta^2 = 0.127$, indicating a marginally lateralized effect. For P2, $F(1,22) = 34.127$, $p < .001$, $\eta_p^2 = 0.608$, suggesting lateralization. Post hoc analyses showed that the P1 amplitude was significantly higher for the contralateral waveform compared to the ipsilateral waveform, $t(22) = 4.235$, $p < .001$, *Cohen's d* = 0.883, $BF_{10} = 91.640$, and the P2 amplitude was significantly lower for the contralateral waveform compared to the ipsilateral waveform, $t(22) = 5.842$, $p < .001$, *Cohen's d* = 1.218, $BF_{10} > 1000$. Similarly, the N1 amplitude was marginal significantly lower for the contralateral waveform compared to the ipsilateral waveform, $t(22) = 1.790$, $p = .087$, *Cohen's d* = 0.373, $BF_{10} = 0.859$ (SFigure 4).

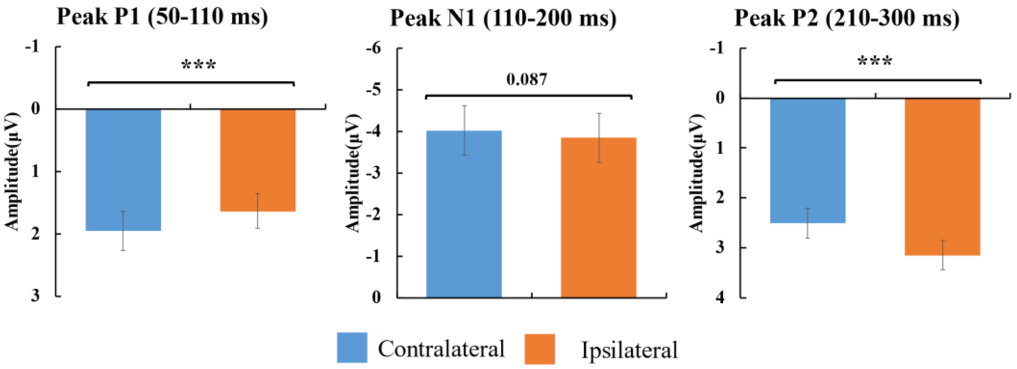

SFigure 4: Peak amplitudes of P1 (50–110 ms), N1 (110–200 ms), and P2 (210–300 ms) for contralateral and ipsilateral waveforms. Error bars represent standard error (SE). *** = p < .001.

Discussion

Overall, these findings confirm that the baseline fluctuations observed in the CDA analysis were indeed driven by ERP components evoked by the arrow cue. We conducted an exploratory analysis using the arrow cue onset as time zero, with a 200 ms pre-cue baseline. This approach allowed us to more clearly define the temporal dynamics of cue-evoked activity and confirm the role of early visual components in mediating attentional processing. We observed several ERP components in both contralateral and ipsilateral waveforms, including a P1 (50–110 ms), N1 (110–200 ms), and P2 (210–300 ms). In the lateralized difference waveforms, an N2pc component was observed from approximately 200–290 ms after the arrow cue onset. Notably, while P1 and N1 reflect early perceptual process and attentional enhancements, the presence of a significant P2 component suggests an additional stage of visual processing, potentially linked to higher-order perceptual organization or decision-related processes.

These results further support the notion that the cue modulated early visual processing (P1/N1) and exerted a selective attentional influence at a later stage (N2pc). The significant main effects for P1 and N1 suggest that the cue enhanced visual attention to specific spatial locations, potentially facilitating target selection in the subsequent memory task. The presence of a P2 component, which was larger in the ipsilateral waveform compared to the contralateral waveform, indicates a possible shift in processing dynamics, potentially reflecting top-down modulation or re-evaluation of the attended location. The robust N2pc effect further highlights the cue's role in attentional resource allocation, reinforcing the idea that spatial cues direct visual attention processes before the onset of memory array.

3 Analyses of the whole CDA amplitude

In the main text, our analyses focused on early (500–650 ms) and late (700–850 ms) CDA components to delineate the distinct contributions of early and late maintenance processes in VWM. To provide a complementary perspective, we also calculated the whole CDA amplitude across the entire measurement time window (500–850 ms). This analysis of the whole CDA amplitude aimed to offer additional insights into the overall neural activity associated with maintaining memory representations under different memory conditions (all-same, partial-same, and all-different). By examining the integrated CDA response, we sought to determine whether the facilitative effects of identical objects on VWM resource consumption were sustained throughout the maintenance period and how these effects compared to those observed in specific temporal windows.

Data analysis

The preprocessing steps and the calculation of difference waveforms for CDA components were described in the main text. The whole CDA amplitude represents the mean amplitude of the CDA component over the entire measurement time window (500–850 ms).

Result

The averaged difference waveforms and CDA results for memory array condition are presented in SFigure 5. The ANOVA revealed significant main effect of the size condition, $F\ (2,44)$ = 4.267, p = .020, η^2_p = 0.162.

Planned pairwise comparisons revealed that the CDA amplitudes were significant difference between the all-different condition (-1.547 ± 0.269 μV) and the all-same condition (-1.174 ± 0.277 μV), $t\ (22)$ =2.373, p = .027, *Cohen's d* = 0.495, BF_{10} = 2.175. Additionally, the CDA amplitudes showed significant difference between the partial-same condition (-1.551 ± 0.232 μV) and the all-same condition, $t\ (22)$ = 2.434, p = .023, *Cohen's d* = 0.508, BF_{10} = 2.421. However, no significant differences were observed between the partial-same condition and the all-different condition, $t\ (22) = 0.031$, p = .976, *Cohen's d* = 0.006, BF_{10} = 0.219.

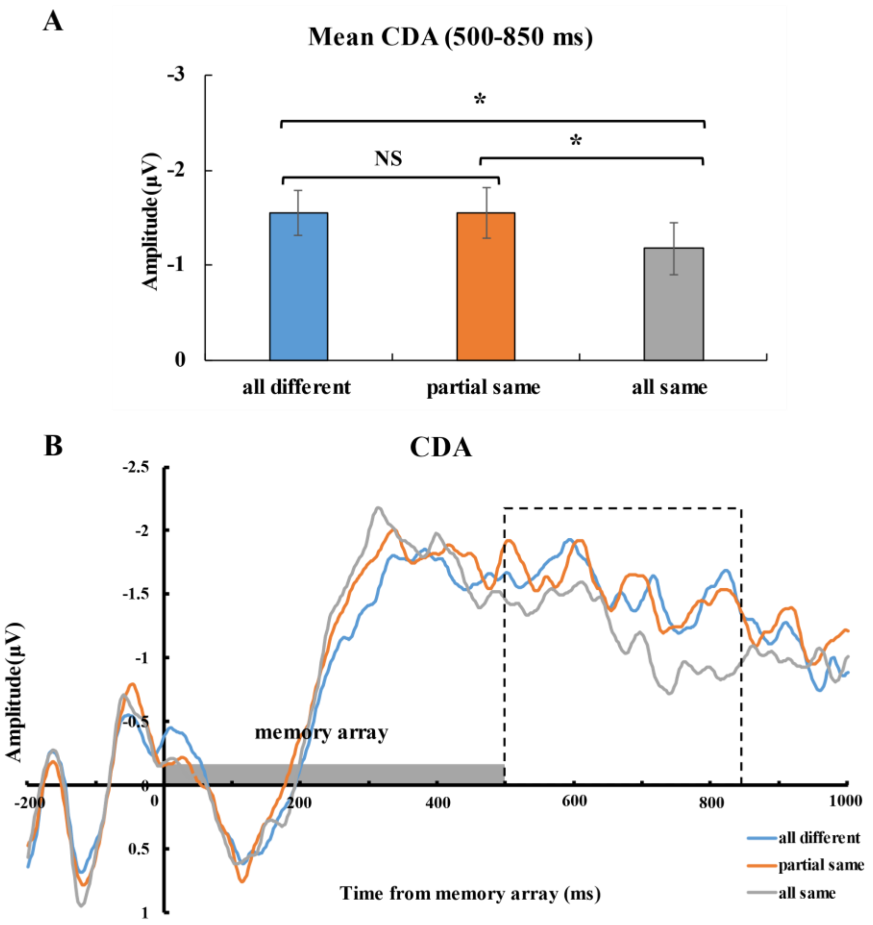

SFigure 5: (A): whole-time window CDA results for each condition. Mean and standard error of the mean for the CDA (500-850 ms) under different memory array conditions (all-same condition, partial-same condition, and all-different condition). Error bars indicate SE. N.S. = no significant difference, * = p < .05. (B): Mean waveforms of the average ERP for different memory array conditions: all-same condition (gray), partial-same condition (orange), and all-different condition (blue), showing the difference waveform (contralateral minus ipsilateral). The waveforms are time-locked to the onset of the memory array (y-axis at time zero). The shaded grey box represents the time window of the memory array presentation.

Discussion

The whole CDA amplitude for the all-same condition was significantly lower than that for the partial-same and all-different conditions, while no significant differences were found between the partial-same and all-different conditions. This finding suggests that under the all-same condition, participants efficiently reduced VWM resource consumption, consistent with the pattern observed for the late CDA component in the main text.

Considering that the analyses in the main text revealed no significant differences between the all-same, partial-same, and all-different conditions during the early

maintenance phase, the effects observed in the whole-time window (500–850 ms) were likely driven predominantly by contributions from the late maintenance phase.

While analyzing the whole CDA amplitude provides an opportunity to evaluate the consistency and robustness of these effects over the entire maintenance period, this approach lacks the temporal resolution to capture the dynamic changes in VWM resource allocation during the maintenance phase. In contrast, the division of CDA components into early and late phases in the main text allowed for a more detailed tracking of these changes. Consequently, we chose not to report the whole CDA amplitude analysis in detail in the main text.

4 Behavioral analysis of sub-conditions within the partial-same condition

In the main text, we analyzed the overall behavioral performance across three primary memory conditions: all-same, partial-same, and all-different. While the partial-same condition demonstrated intermediate performance between the all-same and all-different conditions, it remains unclear whether spatial configurations of identical items within the partial-same condition contribute to observed performance differences. Specifically, the partial-same condition includes arrays where two pairs of identical items are distributed on one visual hemifield, but the spatial arrangement of these pairs may influence VWM performance. To further investigate this possibility, we categorized the partial-same condition into three distinct sub-conditions (examples see SFigure 6) based on the spatial configuration of identical items: (1) Separated-same condition: In this configuration, each pair of identical orientations is separated by an intervening item, meaning no identical items are adjacent. This arrangement likely increases the difficulty of grouping identical items, as spatial separation disrupts perceptual organization; (2) Central-peripheral-same condition: Here, one pair of identical orientations is positioned in central locations within the memory array, while the other pair is placed in peripheral positions. In this configuration, one pair of identical items is adjacent, potentially facilitating partial grouping; (3) Adjacent-same condition: In this configuration, both pairs of identical orientations are adjacent within the memory array. This arrangement provides optimal spatial proximity for grouping, which may enhance perceptual organization and memory performance.

This sub-condition analysis aims to evaluate whether the spatial arrangement of identical items influences VWM performance within the partial-same condition. By comparing behavioral performance (Cowan's K) across these sub-conditions, we seek to clarify the extent to which spatial configurations impact the integration of identical items into memory representations. Furthermore, to contextualize the results, we compare each sub-condition to the all-same and all-different conditions to determine the relative benefits of spatial proximity and grouping within the partial-same condition.

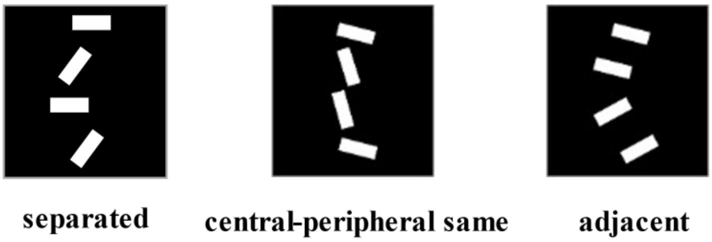

SFigure 6: The three sub-conditions of the "partial-same" condition, including (left) separated-same condition: two identical items are separated by intervening items, (middle) central-peripheral-same condition: two central items are identical, along with two identical peripheral items, and (right) adjacent-same condition: two identical items are adjacent to each other.

Data analysis

To examine the effect of spatial configuration on VWM performance, we first conducted a one-way repeated-measures ANOVA on K values, with sub-condition (separated-same, central-peripheral-same, adjacent-same) as the within-subject factor. Planned pairwise comparisons were conducted using two-tailed paired t-tests to compare differences between sub-conditions (separated-same vs. central-peripheral-same, separated-same vs. adjacent-same, central-peripheral-same vs. adjacent-same).

Next, we performed a broader analysis to compare each sub-condition to the other memory conditions (all-different and all-same). A one-way repeated-measures ANOVA was conducted to examine the effect of memory condition (all-different, separated-same, central-peripheral-same, adjacent-same, all-same) on K values. Planned pairwise comparisons were conducted using two-tailed paired t-tests to compare each sub-condition with the all-different and all-same conditions.

Result

The averaged difference K values for separated-same condition, central-peripheral-same condition, and adjacent-same condition are presented in SFigure 7. The ANOVA revealed a significant main effect of the size condition, $F(2, 44) = 8.86$, $p < .001$, $\eta_p^2 = 0.287$.

Planned pairwise comparisons showed that the K values were significantly different between the separated-same condition (1.742 ± 0.128 items) and the central-peripheral-same condition, $t(22) = 2.441$, $p = .023$, *Cohen's d* = 0.509, $BF_{10} = 2.450$. Additionally, the K values were significantly different between the separated-same condition (1.424 ± 0.117 items) and the adjacent-same condition (1.951 ± 0.101 items), $t(22) = 4.000$, $p < .001$, *Cohen's d* = 0.834, $BF_{10} = 55.267$. A marginally significant difference was also observed between the central-peripheral-same condition and the adjacent-same condition, $t(22) = 1.808$, $p = .084$, *Cohen's d* = 0.377, $BF_{10} = 0.882$.

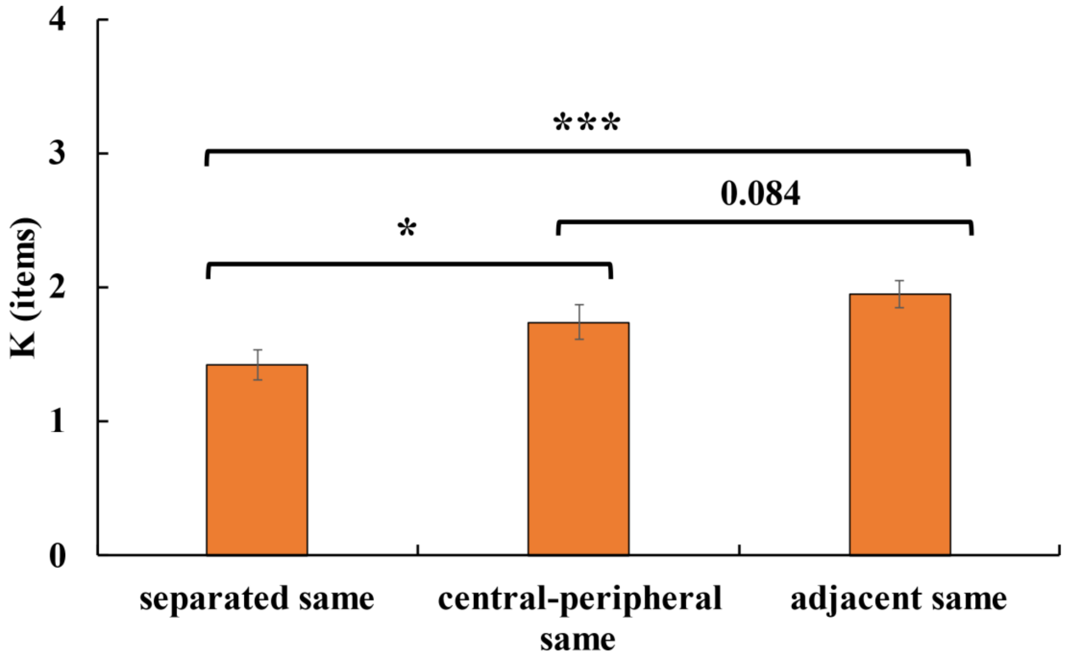

SFigure 7: Cowan's K results for each partial-same sub-condition. Bars represent the mean K values, and error bars indicate the standard error of the mean (SE). Significant differences are denoted as follows: *= $p < .05$, *** = $p < .001$.

Next, we conducted pairwise comparisons between the three partial-same sub-conditions and both the all-same and all-different conditions. The results revealed that the K values for all three partial-same sub-conditions were significantly lower than those for the all-same condition (separated-same: $t(22)$ = 11.841, p < .001, *Cohen's d* = 2.469, BF_{10} > 1000; central-peripheral-same: $t(22)$ = 7.735, p < .001, *Cohen's d* = 1.613, BF_{10} > 1000; adjacent-same: $t(22)$ = 7.051, p < .001, *Cohen's d* = 1.470, BF_{10} > 1000). In contrast, the K values for all three partial-same sub-conditions were significantly higher than those for the all-different condition (separated-same: $t(22)$ = 2.203, p = .038, *Cohen's d* = 0.459, BF_{10} = 1.631; central-peripheral-same: $t(22)$ = 5.511, p < .001, *Cohen's d* = 1.149, BF_{10} > 1000; adjacent-same: $t(22)$ = 6.809, p < .001, *Cohen's d* = 1.420, BF_{10} > 1000) (in SFigure 8).

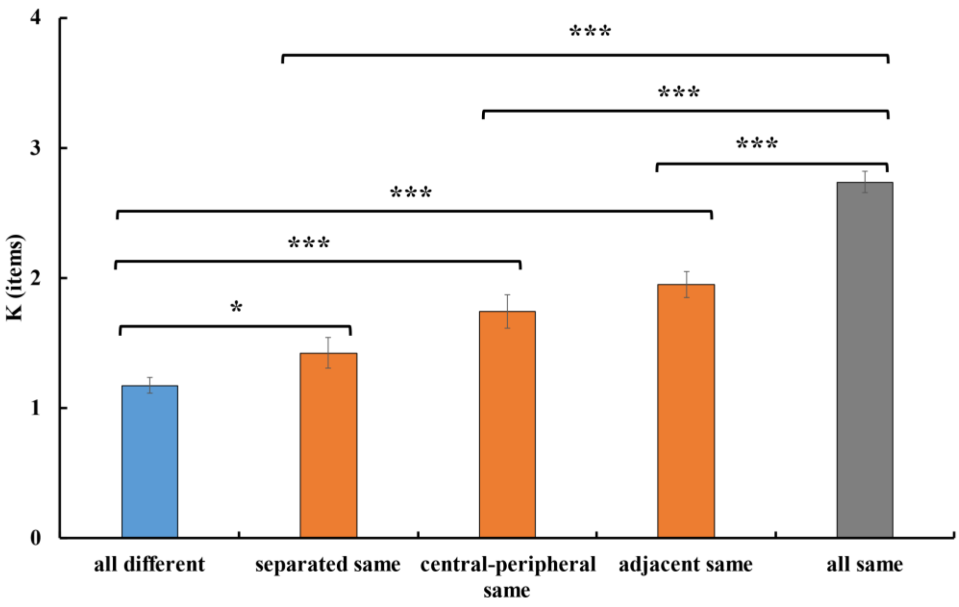

SFigure 8: Cowan's K results for each partial-same sub-condition, the all-same condition, and the all-different condition. Bars represent the mean K values, and error bars indicate the standard error of the mean (SE). Significant differences are denoted as follows: * = $p < .05$, *** = $p < .001$.

Discussion

Our results showed that VWM performance was highest in the adjacent condition and lowest in the separated-same condition. These findings support the hypothesis that the spatial configuration of identical items strongly influences memory performance, with spatial proximity enhancing perceptual grouping and subsequent memory benefits. Notably, even in the separated-same condition, which showed the lowest performance among the sub-conditions, K values were significantly higher than those in the all-different condition. This suggests that participants were able to detect and use the presence of identical items to some extent, even when they were spatially separated. Interestingly, this result contrasts with previous findings suggesting that non-adjacent identical objects provide no memory benefit [3]. A potential explanation for this discrepancy is the consistent placement of memory items within a single visual hemifield in our study, which may have facilitated their integration despite spatial separation.

Taken together with ERP findings from the main text, which revealed no significant differences in CDA amplitudes between the partial-same and all-different conditions, our results suggest that the memory benefit arising from spatial proximity is unlikely to be due to resource reallocation during the maintenance phase of VWM. Instead, this benefit may primarily occur during the retrieval and comparison phases of VWM. Future studies could further investigate this hypothesis by using ERP techniques to examine participants' cognitive processing following the presentation of the probe array.

References

1 Cowan, N. The magical number 4 in short-term memory: A reconsideration of mental storage capacity. *The Behavioral and brain sciences* **24**, 87-185, doi:10.1017/S0140525X01003922 (2001).

2 Rouder, J. N., Speckman, P. L., Sun, D., Morey, R. D. & Iverson, G. Bayesian t tests for accepting and rejecting the null hypothesis. *Psychonomic bulletin & review* **16**, 225-237, doi:10.3758/PBR.16.2.225 (2009).

3 Peterson, D. J. & Berryhill, M. E. The Gestalt principle of similarity benefits visual working memory. *Psychonomic bulletin & review* **20**, 1282-1289, doi:10.3758/s13423-013-0460-x (2013).
